# Vulnerability assessment of English and Welsh coastal areas

**DOI:** 10.1038/s41598-024-78238-0

**Published:** 2024-11-10

**Authors:** Komali Kantamaneni, Liuchang Xing, Vijaya Gupta, Luiza C. Campos

**Affiliations:** 1 United Nations-SPIDER-UK Regional Support Office, Preston, UK; 2 University of Central Lancashire, Preston, UK; 3 University College London, London, UK; 4 Indian Institute of Management (IIM), Mumbai, India

**Keywords:** Coastal vulnerability risk assessment, Physical parameters, Economic parameters, Combined index, Natural hazards, Environmental sciences

## Abstract

The escalating threat of climate change has placed global coastal communities at risk, with rising sea levels and intensified storm events presenting unprecedented challenges. Coastal vulnerability assessments, conducted every 3–5 years, are crucial. This empirical study assesses the Coastal Vulnerability Index (CVI) for the distinct coastal contexts of Dawlish, Happisburgh (England), and Aberystwyth (Wales). The CVI method consists of the Physical Coastal Vulnerability Index (PCVI) and the Economic Coastal Vulnerability Index (ECVI), which provide a multidimensional assessment of vulnerability for coastal zones. This integrated index allows for a nuanced evaluation of vulnerability, distinguishing between sites based on various factors. Additionally, this study conducted a correlation analysis to understand the associations between the parameters. The findings demonstrate that physical features like beach and dune widths significantly impact a location’s natural defences, and economic factors such as property values and population density are equally crucial in determining societal risks and potential financial repercussions. The Combined Coastal Vulnerability Index (CCVI) results confirm the effectiveness of incorporating a diverse range of variables. Despite its substantial economic value, it reveals that Dawlish requires targeted protective measures, whereas Happisburgh needs an increased focus on its most vulnerable sectors. Aberystwyth emerges as the area with the highest overall vulnerability, underscoring the need for comprehensive coastal management practices. The study’s conclusions emphasize the essential role of adaptive, integrated management strategies in enhancing coastal resilience against the complex threats posed by climate dynamics. Moving forward, the indices established herein advocate for their use in strategic planning and policymaking to strengthen coastal regions in the face of sea-level rise and climatic variability. This investigation lays the groundwork for future research, aimed at refining and expanding these methodologies, aspiring to develop a detailed national coastal vulnerability atlas, a critical tool for informed decision-making and safeguarding at-risk communities.

## Introduction

Vulnerability refers to the susceptibility to potential harm^1,2^. In the context of natural hazards, vulnerability signifies the likelihood of being adversely affected by such events. Certain individuals and locations exhibit higher vulnerability to specific hazards compared to others^3,4^. Existing literature shows that few studies have undertaken both physical and economic vulnerability risk assessments in the UK. However, there is a notable absence of corresponding research on socio-economic vulnerability^5^. The coastal region serves as a critical interface between land and sea, playing a pivotal role in ecological balance, biodiversity conservation, economic activities, and as a frontline defence against the impacts of climate change^6,7^. The UK coastline spans 17,381 km, with 3008 km (17.3%) currently eroding. England is the most affected region, with 29.8% of its coastline experiencing erosion^8^. Despite its essential ecological and socio-economic functions, the coastal region faces increasing vulnerability to climate change^9–12^. Climate change poses a critical threat to regions worldwide, especially coastal areas. Its exacerbation of extreme events comes with a staggering annual cost of £108 billion^13^. With almost 44% of the global population living within 150 km of the coast and eight of the ten largest cities in the world positioned near the coastline, it is clear that coastal areas play a critical role in our lives^14^. As global temperatures continue to rise^15–18^, urgent attention is required to implement adaptation and mitigation strategies to safeguard the environment and the livelihoods of those dependent on these invaluable areas. Coastal regions around the world are highly susceptible to natural disasters because of their proximity to the ocean, high population density, and extensive economic activities^19–27^. Ongoing climate change exacerbates these risks, with events like sea-level rise, heavy rainfall, and cyclones posing continuous threats to the well-being of coastal inhabitants, infrastructure, and the surrounding ecosystems^28–31^.

As global interdependence continues to expand through economic, social, and cultural integration, the interconnected nature of nations makes it unavoidable that consequences originating in one country or region will inevitably spread to other parts of the world, including the United Kingdom (UK)^32^. The UK boasts the longest coastline in Europe, measuring 17,381 km, and 17% of the UK coastline is currently experiencing the impact of erosion^33^. Compared to the other three administrative regions (Wales, Scotland, and Northern Ireland), England is considered overall more vulnerable to sea-level rise and flooding^8,34–37^. With an increased rate of sea-level rise, the coastal region will be continually exposed to heightened rates of erosion, flooding, and fluctuations in weather conditions^35,38,39^. Moreover, the vulnerability of specific coastal regions needs to be determined by site-specific factors such as topography, landscape, geology, coastal hazards, and climate change, among other elements^40,41^.

The Coastal Vulnerability Index (CVI), originally developed by Gornitz^42^ in 1990 with a quantitative scale from 1 to 5, has become one of the most widely accepted methodologies in coastal vulnerability studies. Since its development, researchers have adapted the model by modifying indicator parameters and applying it to various regions^43–56^.The CVI is crucial for assessing vulnerability across multiple dimensions, providing a standardized framework for comparing vulnerability assessments across different areas. There is extensive literature on the development of CVI using diverse parameters to evaluate coastal segments globally, as illustrated by the examples in Table 1. Despite its widespread use at regional, national, and international levels, a gap remains in its applicability to different geographical contexts with diverse physical and economic parameters.

While significant progress has been made in developing approaches to assess coastal vulnerability, most studies have focused primarily on either physical or economic factors. Kantamaneni et al.^5^ introduced an innovative approach with the Combined Coastal Vulnerability Index (CCVI), which produces a quantified index that allows for comparative evaluations of vulnerability across regions. This approach is based on earlier models that have been successfully applied to different geographical areas, demonstrating their robustness^57–60^. The methodology was originally designed for coastal vulnerability assessments in England and Wales and was first applied to Dawlish, Aberystwyth, and Happisburgh in 2016. These areas are particularly vulnerable to climate change and related hazards, such as coastal flooding, erosion, high waves, storm surges, and sea level rise, despite the presence of coastal defence structures^61–65^.

**Table 1:** Coastal vulnerability assessments and parameters used for diverse global geographical locations in the last 10 years (2015–2024).

Coastal vulnerability Index (CVI)	Location	Parameters/indicators
Multi-criteria evaluation approach to coastal vulnerability index development in micro-tidal low-lying areas^48^	Lithuania, Europe	Historical shoreline change rate, Beach width and height, Underwater slope and sand bars, Beach sediments, Mean significant wave height
Coastal vulnerability assessment of the predicted sea level rise in the coastal zone of Krishna–Godavari delta region, Andhra Pradesh, east coast of India^66^	Andhra Pradesh, India (Asia)	Regional slope, coastal elevation, geomorphology, significant wave height, mean tidal range and relative sea level
Assessment of vulnerability for coastal erosion with GIS and AHP techniques case study: Southern coastline of Sri Lanka^67^	Southern coastline of Sri Lanka, (Asia)	Slope, geomorphology, erosion rate, dune width, tide direction, tidal range, wave height
Assessing coastal vulnerability: Development of a combined physical and economic index^5^	United Kingdom	Beach and dune width, coastal slope, distance of vegetation, distance of building behind the back beach, rocky outcrop, sea defences, commercial and residential properties, economic value of site, population, coastal erosion and flood impact
Assessment of the Coastal Vulnerability Index in an Area of Complex Geological Conditions on the Krk Island, Northeast Adriatic Sea^68^	Croatian Eastern Adriatic Coast, Europe	Coastal slope, beach width, and significant wave height
Assessment of the coastal vulnerability to sea level rise: Sultanate of Oman^69^	Sultanate of Oman, Arabia	Coastal geomorphology, elevation, slope, tidal range and bathymetry of the nearshore zone
Development of a Multi-Dimensional Coastal Vulnerability Index: Assessing vulnerability to inundation scenarios in the Italian coast^70^	Italian coast, Europe	Elevation, distance from coastline, shoreline evolution trend, sensible segments of the population, GDP, land use patterns
Coastal Vulnerability Assessment: A Case Study of the Nigerian Coastline^71^	Nigeria, Africa	Coastal slope, bathymetry, geomorphology, wave height, mean tidal range, shoreline change rate and relative sea-level rise, population, cultural heritage, land use/land cover and road network
Toward an Integrated Probabilistic Coastal Vulnerability Assessment: A Novel Copula-Based Vulnerability Index^72^	South Carolina, United States of America	Coastal hazard events, hurricane track density, land use, sea-level rise, surge height, distance from coast, cost of fatalities
An integrated approach to the spatial distribution of the coastal infrastructure vulnerability by using coastal vulnerability index and hot spot analysis: a case study of Kusadasi-Selcuk^73^	Kusadasi-Selcuk, Turkey	Geomorphology, coastal slope, relief, mean seal level, mean tide range, mean wave high, shoreline erosion and accretion

Given their high vulnerability, it is crucial to reassess these areas every 3–5 years to accurately track changes in risk levels. To our knowledge, this study is the only assessment over the past eight years that examines both the physical and economic vulnerability of these specific case study areas, making it unique. Therefore, the present study evaluates the vulnerability of Dawlish, Aberystwyth, and Happisburgh, which were initially identified through existing literature, recent disaster events, and coastal site visits. Moreover, CCVI indices can serve as benchmarks for regional governments, helping them develop and implement strategies to mitigate coastal vulnerability and reduce the impacts of coastal disasters on towns, populations, and economies.

## Study areas

**Fig. 1:**
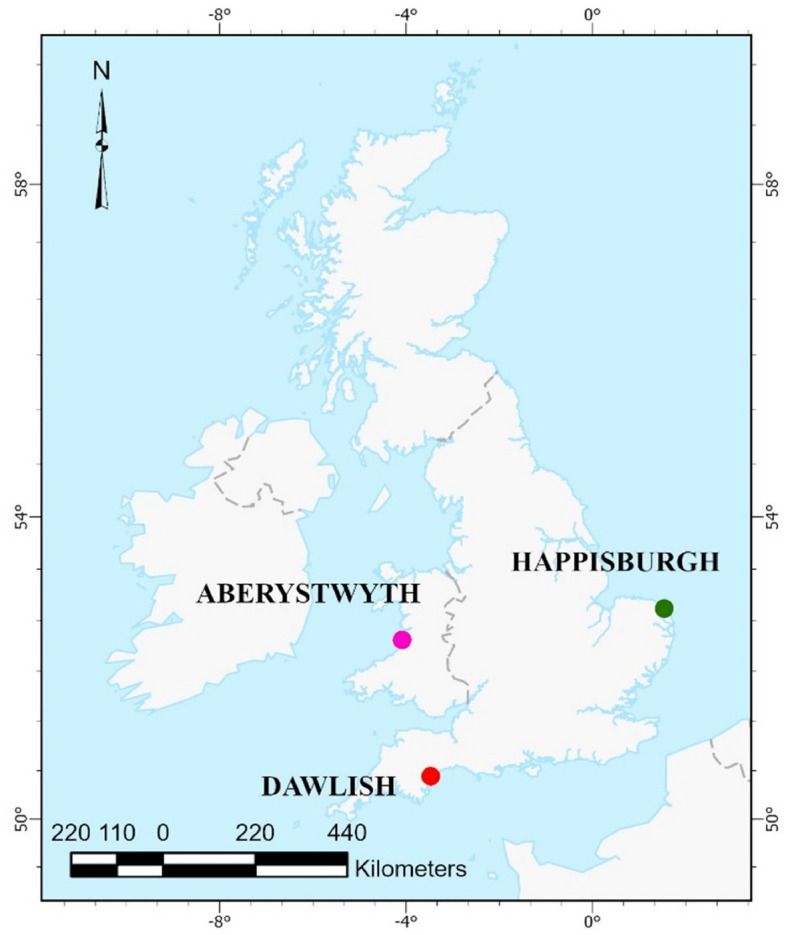
Case study area map. *Source* Second author created by ArcGIS 10.3.1 version.

Recent coastal disasters, existing literature, and several visits to the coast were used to identify the most and least suitable areas with varying geological, physical, and socio-economic features. Initially, several areas were considered, but due to time and financial limitations, the selection was narrowed down to three. In this research, three UK regions previously identified^5^ with significant coastal vulnerability were selected for accessing: Dawlish (England), Aberystwyth (Wales), and Happisburgh (England) (Fig. 1). To supplement data and insights, not available through maps and existing datasets, field investigations were carried out in Dawlish and Aberystwyth. However, logistical constraints, such as time and distance, precluded a similar investigation in Happisburgh. The findings and observations from these field visits are elaborated upon in the discussion section, offering ground-level perspectives on coastal vulnerability in these areas.

### Dawlish

**Fig. 2:**
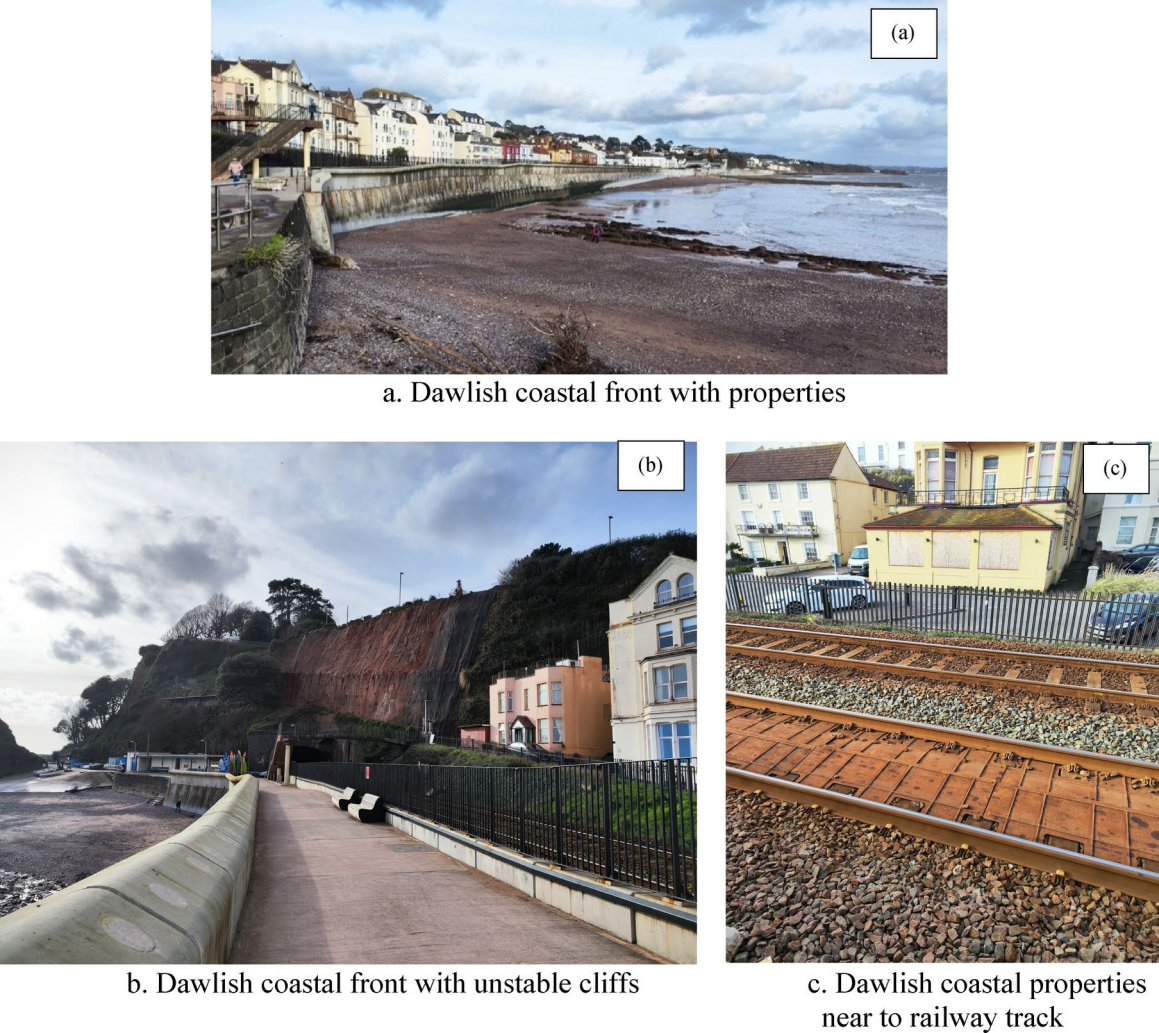
Photographs were taken by the second author in Feb 2024 during the data collection.

Dawlish, a seaside resort town in Teignbridge on the south coast of Devon, England (Fig. 2a–c), exemplifies the acute vulnerability of coastal communities to climate change and extreme weather events. As of 2021, Dawlish had a population of 15,257 and has evolved from a small fishing port into a popular tourist destination, experiencing significant seasonal population increases^74^. This growth highlights the importance of resilient coastal defences, particularly in light of the February 2014 flood event, which severely damaged critical railway infrastructure^74–76^. The event underscored the complexities of coastal defences and their economic impacts, leading to a two-month rail closure and an estimated £1.2 billion in economic losses^38^.

In response, Dawlish has undertaken substantial enhancements to its coastal defences, including constructing a taller seawall. This new seawall was designed using historical data, eyewitness accounts, and advanced modelling to fortify against future threats^77^. Following this event, substantial efforts were directed towards enhancing the resilience of coastal defences to mitigate future overtopping and erosion risks. These measures are part of a broader strategy aimed at fortifying the coastal infrastructure against anticipated increases in storm severity and frequency, reflecting a shift towards more sustainable and resilient coastal management practices. Further analysis highlights the importance of incorporating historical data, eyewitness accounts, and advanced modelling techniques in developing strategies to protect coastal infrastructure^77^. Moreover, the ongoing challenges posed by sea-level rise necessitate an assessment of existing coastal defence mechanisms to ensure the long-term protection and sustainability of coastal communities like Dawlish^65^. Together, these efforts exemplify the critical need for integrated, adaptive management approaches in safeguarding vulnerable coastal regions against the multifaceted threats posed by climate change and sea-level rise.

### Aberystwyth

**Fig. 3:**
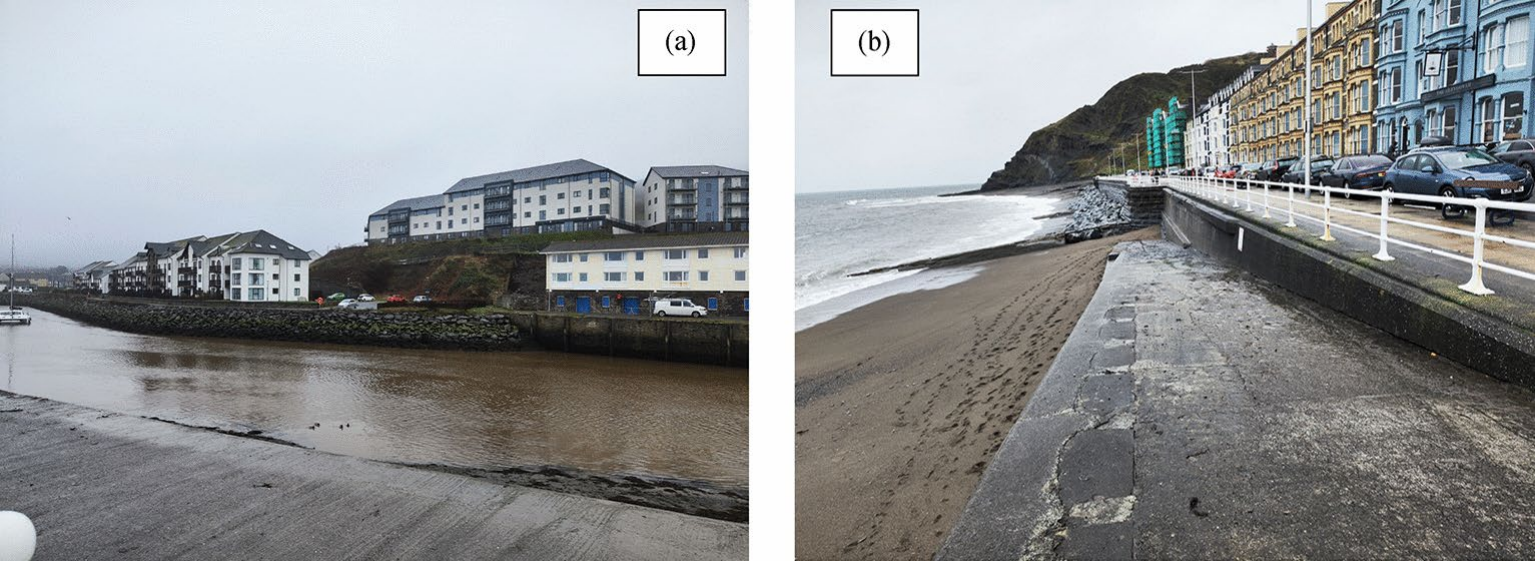
(**a**–**b**) Coastal Infrastructure at Aberystwyth. (Photographs were taken by the second author in Feb 2024 during the data collection).

Aberystwyth, a coastal town in Ceredigion, Wales, is distinguished not only by its natural beauty and historical landmarks but also by its significant vulnerability to coastal erosion (Fig. 3a,b). Situated at the confluence of the Ystwyth and Rheidol rivers and surrounded by hills—Pendinas to the south, Constitution Hill to the north, and Penglais Hill to the east—the town features a harbour, two sandy beaches, castle ruins, and a pier, attracting both visitors and locals alike^78^. However, the same geographical features that contribute to the town’s charm also increase its vulnerability to coastal hazards. The West Wales coastline, including Aberystwyth, faces ongoing challenges due to erosion. Notably, the glacial embayment experienced significant recession rates of up to 0.25 m annually between 1983 and 1985, demonstrating the area’s dynamic and sometimes precarious interaction with natural forces^62^.

### Happisburgh

**Fig. 4:**
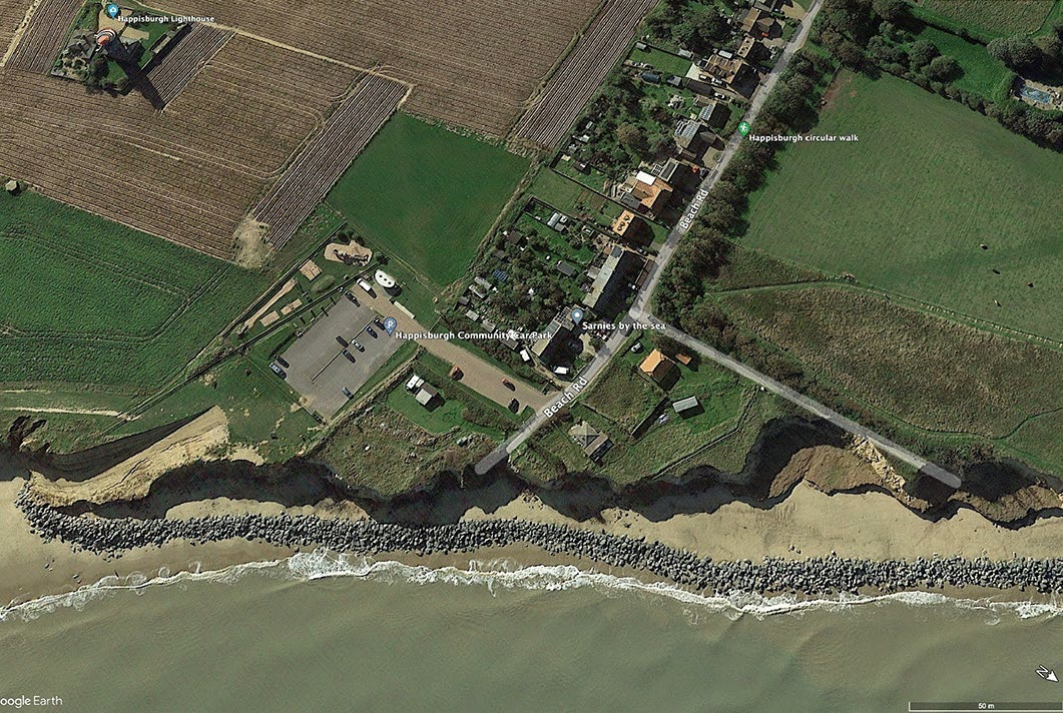
Happisburgh coastal erosion in 2024. *Source* Google earth Pro maps, 2024.

Happisburgh, a village and civil parish located in Norfolk, England, is a poignant example of the dynamic challenges coastal communities face due to erosion and the impacts of human intervention on natural coastal processes (Fig. 4). The village has more than 1400 inhabitants and approximately 600 houses^79^. It has been subject to the forces of nature for millennia, with steadily rising sea levels contributing to the ongoing erosion of the Norfolk coast^65^. In the early 1990s, a significant change occurred in Happisburgh’s coastal management approach when approximately one km of coastal defences was removed^80^. This action triggered a period of rapid erosion, markedly higher than historical rates, underscoring the profound impact of coastal defence structures on shoreline retreat dynamics. Over two decades, the coastline receded by about 140 m, a stark illustration of the accelerated erosion following the removal of man-made barriers^81^.

## Methodology

In this study, we have adapted the Physical Coastal Vulnerability Index (PCVI), Economic Coastal Vulnerability Index (ECVI), and CCVI from Kantamaneni et al.’s methodology to the selected case study sites. Since all these parameters fall under the broader category of existing physical and economic parameters, we used the same terminology to update the CCVI knowledge for Happisburgh, Aberystwyth, and Dawlish.

The integration of PCVI and ECVI allows for a relatively comprehensive assessment of a region’s coastal vulnerability from both physical and economic perspectives. This framework permits adjustment to parameters, measurement methodologies, and the rating system used to convert parameters into the index, aiming to enhance the comprehensiveness of the CCVI. Due to time constraints of this study, only a selection of parameters and specific target areas were researched, necessitating the use of the same measurement methods and rating system.

The field study was conducted from June to July 2024.

### Physical coastal vulnerability index

The physical parameters incorporate Beach Width, Dune Width, Coastal Slope, Distance of Vegetation behind the Back Beach, Distance of Built Structures behind the Back Beach, and Sea Defences, complete with their respective ratings. Notably, the parameter of Rocky Outcrop was excluded (Table 2). This decision was made because its characteristics are already accounted for within the category of Sea Defences, thus eliminating the need to treat it as a distinct parameter in this analysis.

**Table 2:** Physical parameter rating. (*Source* modified from^5^).

No	Physical parameter with symbol	Extremely low (1)	Low (2)	Moderate (3)	High (4)
1	Beach width (a)	> 150 m	100–150 m	50–100 m	< 50 m
2	Dune width (b)	> 150 m	50–100 m	25–50 m	< 25 m
3	Coastal slope (c)	> 12%	12–8%	8–4%	< 4%
4	Distance to built structures behind the back beach (d)	> 600 m	600–200	200–100 m	< 100 m
5	Distance of vegetation behind the back beach (e)	> 600 m	600–200	200–100 m	< 100 m
6	Sea defence (f)	> 50%	20–50%	10–20%	< 10%

### Data

**Fig. 5:**
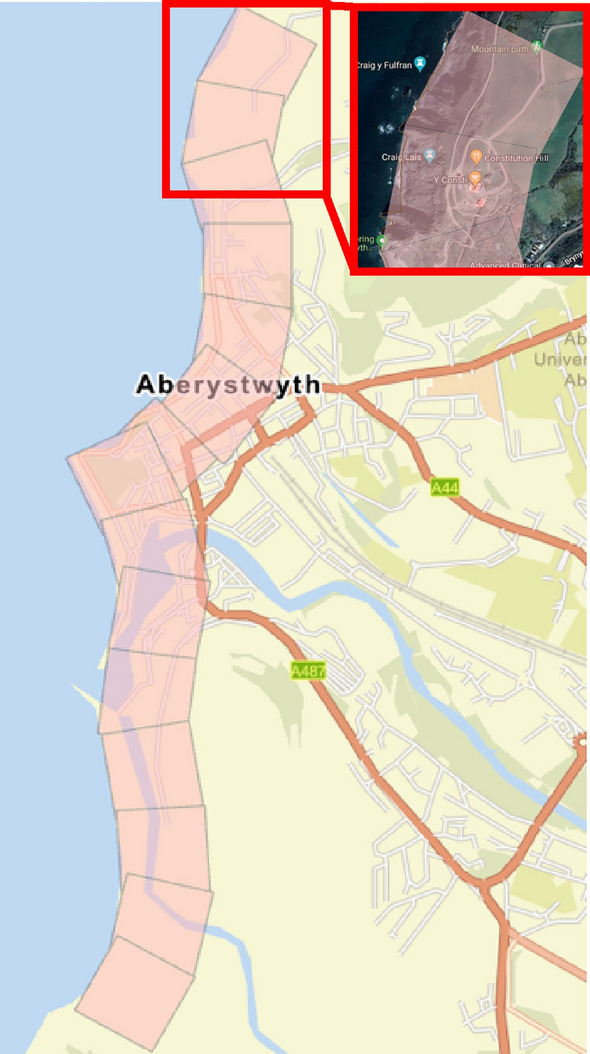
500 m-by-500 m cell in Aberystwyth for physical parameter measurement. *Source* The second author created these pictures by using ArcGIS 10.3.1 version.

Data for the PCVI was collected from various sources. The study area boundaries for Dawlish and Aberystwyth were determined using the Boundary and Location Data (Boundary line) provided by Digimap’s Ordnance Survey. Happisburgh’s boundary definition was derived from the local village’s official website. After delineating the research boundaries, grids consisting of 500 m-by-500 m cells (Fig. 5) were established along the coastline of each area using ArcGIS Pro, designated as the valid research zone for each region. Data collection was then conducted along a baseline established by extending a line perpendicular to the coastline from the inward midpoint of each cell’s coastal edge. This approach ensured that all collected data were one-dimensional, anchored to this defined baseline.

The physical cell dimensions are determined by the need for high spatial resolution to accurately capture the variability of coastal processes. Physical parameters such as beach width, dune width, and coastal slope can change significantly over short distances, and using a finer cell size ensures that these variations are accurately reflected in the PCVI.

The formula for calculating the PCVI for each cell is shown in Eq. (1).

1 $$\text{PCVI}={\text{P}}_{\text{a}}+{\text{P}}_{\text{b}}+{\text{P}}_{\text{c}}+{\text{P}}_{\text{d}}+{\text{P}}_{\text{e}}+{\text{P}}_{\text{f}}$$

where, ${P}_{\text{a}}$ to ${\text{P}}_{\text{f}}$ represents the rating of each physical parameter with their designated symbols from a to f.

Based on Table 3, the values were allocated as follows:

**Table 3:** Vulnerability categories of PCVI.

Physical vulnerability	
Physical vulnerability score	Level of vulnerability
< 6	Extremely low
6–12	Low
13–18	Moderate
19–24	High

If the PCVI = 1 + 1 + 1 + 1 + 1 + 1 = 6

Minimum level of total CVI score is 6

If the PCVI = 4 + 4 + 4 + 4 + 4 + 4 = 24

Maximum level of score is 24

Then, cumulatively, all scores will be summed based on the final PCVI scores.

### Economic coastal vulnerability index

**Fig. 6:**
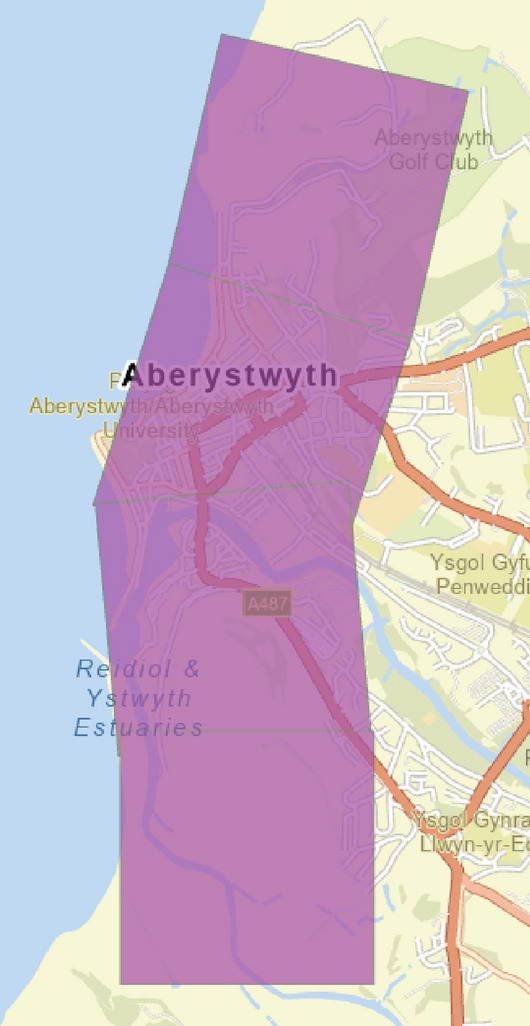
1 km-by-1 km cell in Aberystwyth for economic parameter measurement. *Source* The second author created these pictures by using ArcGIS 10.3.1 version.

The selection of economic parameters incorporates Commercial Properties, Residential Properties, the Economic Value of the Site, and Population, along with their corresponding ratings (Table 4). The boundaries for each region remain consistent with those defined in section “Physical coastal vulnerability index”, but the research areas are now demarcated by 1 km-by-1 km cells (Fig. 6). Unlike the approach for Physical Parameters, which focuses on one-dimensional data collected along a single baseline, the Economic Parameters consider the aggregate data within each 1 km-by-1 km cell, emphasizing a comprehensive assessment of the area’s economic and demographic characteristics.

**Table 4:** Economic parameter ratings and corresponding vulnerability levels. *Source* modified from^5,74,82^.

No	Economic parameter with symbol	Extremely low (1)	Low (2)	Moderate (3)	High (4)	Extremely high (5)
1	Commercial properties (a)	< 2 m	2–10 m	> 10–30 m	> 30–70 m	> 70 m
2	Residential properties (b)	< 30 m	30–80 m	> 80–130 m	> 130–180 m	> 180 m
3	Economic value of the site (c)	< 10 m	10–50 m	> 50–100 m	> 100–150 m	> 150 m
4	Population (d)	< 500	500–2000	> 2000–5000	> 500–10,000	> 10,000

*m* millions.

The Economic Cell dimension aligns with the broader spatial scale at which economic factors operate. Economic data, including property values, population density, and infrastructure, are often aggregated over larger areas, making a 1 km^2^ cell size appropriate for capturing these variables. This larger cell size ensures that the ECVI reflects significant economic conditions across a broader area, rather than focusing on micro-level variations that may not substantially impact overall vulnerability. Additionally, it facilitates the integration of economic data with existing administrative and statistical datasets, enhancing the accuracy and applicability of the assessment.

The formula for calculating the ECVI for each cell is shown in Eq. (2).

2 $$\text{ECVI}={\text{E}}_{\text{a}}+{\text{E}}_{\text{b}}+{\text{E}}_{\text{c}}+{\text{E}}_{\text{d}}$$

where, ${\text{E}}_{\text{a}}$ to ${\text{E}}_{\text{d}}$ represents the rating of each economic parameter with their designated symbols from a to d.

Based on Table 5, the values were allocated as follows:

**Table 5:** Vulnerability categories of ECVI.

Economic vulnerability	
Economical vulnerability score	Level of vulnerability
< 4	Extremely low
4–8	Low
9–12	Moderate
13–16	High
17–20	Very high

If the ECVI = 1 + 1 + 1 + 1 = 4

Minimum level of total CVI score is 4

If the ECVI = 5 + 5 + 5 + 5 = 20

Maximum level of score is 20

Then, cumulatively, all scores will be summed based on the final ECVI scores.

### Combined coastal vulnerability index

The CCVI is formulated by integrating the PCVI and the ECVI. This combination offers a more holistic approach to assessing coastal regions, rather than focusing on isolated segments. By merging these indices, the CCVI provides a comprehensive method to evaluate the broader vulnerabilities of coastal areas.

3 $$CCVI=\frac{\frac{\left(\sum PCVI\right)}{N}+\frac{\left(\sum ECVI\right)}{N}}{2}$$

where N is the number of cells for PCVI and ECVI.

### Correlation of parameters

In addition to analysing the PCVI, ECVI, and CCVI, this study also performed a correlation analysis using Stata 14 to establish the links between various parameters. The correlation test encompassed both physical and economic parameters.

### Physical parameters and its measurement

#### Beach width

**Fig. 7:**
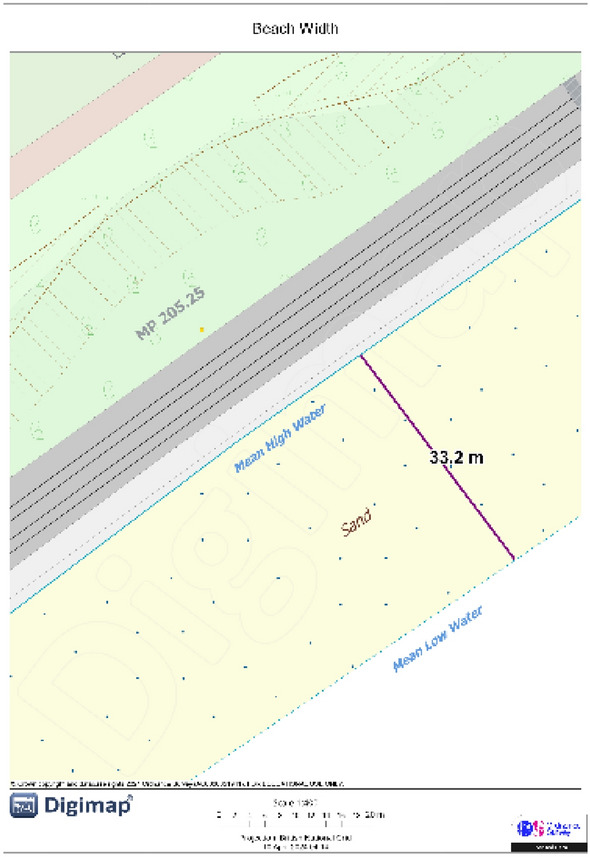
Beach width measurement on OS Digimap. *Source* These pictures were created by the second author using ArcGIS 10.3.1 version on the university’s (University College London-UCL) licensed version of ordnance survey and Digimap.

The inherent sensitivity of soft sedimentary coasts, particularly beaches, to environmental changes is highlighted by their rapid and dynamic adjustment to prevailing conditions, such as sea level fluctuations and variations in sediment supply. Historical events, such as mid-Holocene sediment switching, highlight the complex interplay between relative sea level changes and sediment availability, directly influencing beach width and coastal erosion patterns^83^. These factors make beach width a critical parameter for understanding and mitigating coastal vulnerability. For this project, the Beach Width measurement (Fig. 7) is carried out using OS Digimap. After importing the cell shapefile for each region, the distance between the Mean Low Water line and the back beach on the middle baseline will be measured in meters to determine the Beach Width for that cell.

#### Dune width

**Fig. 8:**
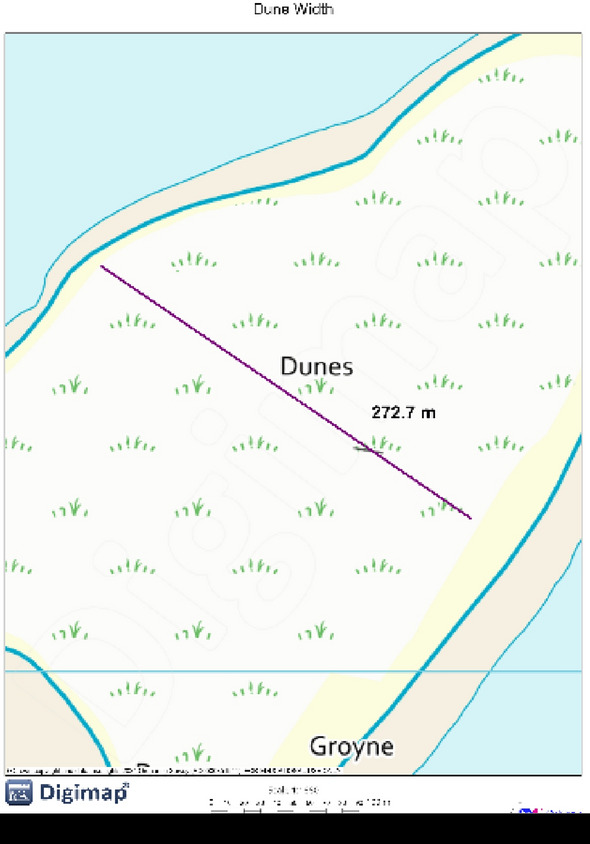
Dune Width measurement on OS Digimap. *Source* These pictures were created by the second author using ArcGIS 10.3.1 version on the university’s (University College London-UCL) licensed version of ordnance survey and Digimap.

Dune width is a critical parameter in coastal protection, serving as a natural barrier against storm surges and wave impacts, thus reducing the risks of coastal erosion and flooding. Extensive dune systems also regulate coastal groundwater by supporting a freshwater lens, playing a pivotal role in preventing saltwater intrusion and maintaining ecological balance. These systems, especially when wide, provide enhanced defence against the forces of nature by stabilizing sand and buffering the coastline against the impacts of sea-level rise and extreme weather events^84,85^. The process for measuring Dune Width is similar to that for Beach Width (Fig. 8). The total length of the dune in meters is measured via OS Digimap along the middle baseline for each cell.

#### Coastal slope

The coastal slope is instrumental in determining the vulnerability of coastal regions to erosion, inundation, and sea-level rise, underscoring its significance in coastal vulnerability assessments. Its influence extends to sediment transport dynamics and the effectiveness of coastal defences, where variations can significantly alter erosion rates and the efficiency of wave energy dissipation^86^. To measure the Coastal Slope, Google Earth Pro was employed to visualize the slope through an elevation profile. The Coastal Slope was determined along the middle baseline, marked by a bolded red line, with the average slope value representing the Coastal Slope.

#### Distance of built structures behind the back beach

**Fig. 9:**
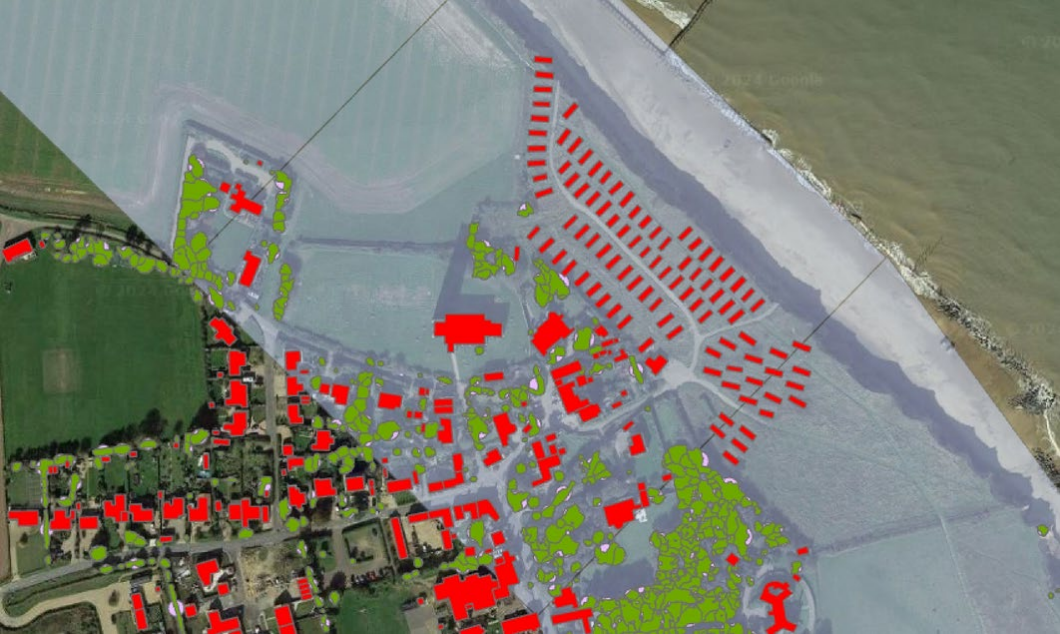
Building height and National Tree Map from OS Digimap on ArcGIS Pro intersected with 500 m-by-500 m cell (Red: Building; Green: Canopy, and Pink: Crown).

The distance to built structures behind the back beach serves as a crucial parameter for understanding and mitigating coastal vulnerability, reflecting the need for strategic infrastructure placement to enhance coastal resilience. This measure gauges the potential risk to human life and property in coastal zones, emphasizing the importance of incorporating adequate buffer zones in coastal planning and development. Furthermore, it aids in assessing the capacity of coastal ecosystems capacity to provide natural defence mechanisms against coastal hazards, ensuring the long-term sustainability and protection of both natural and human-made environments^5^. This physical parameter was measured using ArcGIS Pro, along with Building Height data (Fig. 9) sourced from OS Digimap. This dataset consists of a shapefile that includes all building outlines and their respective heights. For this analysis, the total length of buildings located along the middle baseline was calculated to assess the distance of built structures behind the back beach.

#### Distance of vegetation behind the back beach

The distance of vegetation behind the back beach is a vital parameter for assessing coastal vulnerability, highlighting the role of natural barriers in enhancing shoreline resilience against erosion and storm impacts. By maintaining and measuring this distance, we can gauge the effectiveness of coastal vegetation in providing a natural defence mechanism, crucial for long-term coastal management strategies^5^. It is important to note that this parameter does not include the dunes. The methodology employed for measuring this parameter mirrors that used for previous parameters, utilizing ArcGIS Pro and the National Tree Map from OS Digimap. This resource provides a shapefile detailing the crown and canopy shapes of vegetation. The aggregate length of vegetation (Fig. 9) along the middle baseline is quantified to determine the distance of vegetation behind the back beach.

#### Sea defence

Adapting to climate change by deploying coastal defence structures like seawalls, breakwaters, and groynes is essential for modifying hydrodynamic regimes and protecting coastal zones from erosion and flooding. These structures play a critical role by absorbing wave energy and altering sediment dynamics, thus maintaining the integrity of coastal infrastructure and habitats. The measurement of Sea Defence utilizes ArcGIS Pro, complemented by basemaps from Google Maps. The initial step involves delineating all sea defences, including retaining walls (seawalls), outcrop rocks, and other sea defence types, into a shapefile. Subsequently, the cumulative length of all identified sea defence types is measured. The final step entails calculating the percentage of sea defence coverage. This is done by evaluating the total sea defence length per 100 m of coastal area and then converting this figure into a percentage.

### Economic parameter measurement

#### Commercial properties

The assessment of commercial properties within coastal vulnerability studies provides essential data on economic values at risk, facilitating the quantification of potential financial losses due to coastal hazards. Evaluating the spatial distribution and economic significance of commercial properties aids in prioritizing areas for coastal defence investments, directly impacting policy and resource allocation decisions. Furthermore, this analysis contributes to the development of comprehensive coastal management plans, integrating economic resilience with environmental and infrastructural safeguards^87^.

The approach to measuring Economic Parameters significantly diverges from that of Physical Parameters. While the measurement of Physical Parameters is confined to data collected along a baseline, the Economic Parameter assessment encompasses a comprehensive collection of data relevant to the entire area within each defined cell. Specifically, for the measurement of commercial properties, data from the OS Digimap’s Points of Interest database was utilized. After filtering, several categories related to commercial properties were retained, including Accommodation, Eating and Drinking; Commercial Services; Education and Health; Manufacturing and Production; Retail; and Sport and Entertainment. Subsequent steps involved conducting online market research (such as Zoopla and Rightmove) to determine the average market value of each category within the study area. This process enabled the calculation of the total economic value of commercial properties within each cell, providing a detailed economic assessment integral to understanding the overall economic vulnerability of the coastal regions under study.

#### Residential properties

The significance of residential properties in mitigating coastal vulnerability is highlighted by their direct association with economic valuation and risk exposure in coastal zones. Assessing the distribution and economic value of residential properties allows for a quantified evaluation of potential economic impacts of coastal hazards, helping to identify highly vulnerable areas and prioritize them for intervention. Furthermore, the spatial distribution of residential properties influences the planning and effectiveness of coastal defence mechanisms, as areas with higher concentrations of residences may require more robust protective measures to mitigate the risk of erosion, flooding, and storm surges. These aspects are crucial for developing targeted coastal management strategies that aim to minimize economic losses and ensure the safety and sustainability of coastal communities^87^.

For the measurement of residential properties, the study employed ArcGIS Pro to process data from Digimap, including points of interest and building height information. Buildings that intersected with points of interest were excluded from the residential properties category, treating the remaining structures as residential properties. Subsequently, market research was conducted on online platforms such as home.co.uk to determine the average price of residential properties within each cell^3^. These findings were then compared with House Price Statistics released by the UK government to ensure that the values were within a reasonable range. This process not only validated the market research data but also preserved the unique price characteristics of residential properties within each cell, providing a nuanced understanding of the economic aspects contributing to coastal vulnerability.

#### Economic value of site

The economic value of coastal sites is quantified based on the potential costs of damage or loss due to coastal hazards, incorporating the valuation of infrastructure, residential, and commercial properties at risk. This valuation is crucial for directing coastal management resources towards areas with the highest economic stakes, ensuring that protective measures are economically efficient. Additionally, the economic value serves as a key parameter in coastal vulnerability indices, enabling a prioritized response to threats based on the financial implications of coastal hazards on the built environment and local economies^5^.

The Economic Value of the Site parameter is estimated by subtracting the construction costs from the previously measured values of residential and commercial properties. This approach introduces considerable uncertainty but offers a rough estimate of the site’s economic value. Construction costs are calculated based on unit prices per square meter for various types of buildings as provided by Spon’s Architects and Builders Price Book^88^. Since the type and number of stories of buildings vary across different areas, the calculations must be adjusted according to the characteristics of houses within each cell. While this method is imprecise, it provides a preliminary understanding of the economic valuation of sites.

#### Population

The density and distribution of population in coastal areas are critical factors in assessing and mitigating coastal vulnerability. High population densities complicate evacuation and emergency response efforts, necessitating detailed planning and resource allocation. Accurately measuring coastal populations allows for the identification of high-risk areas, enabling targeted mitigation strategies and the prioritization of resources to enhance resilience and reduce potential impacts from coastal hazards^89^. Population data is derived from Society Digimap’s Population Density, based on the 2011 estimates of the usual resident population in the United Kingdom^5^. This shapefile is imported into ArcGIS Pro, where spatial analysis is conducted to ascertain the specific population within each cell. This method provides relatively accurate data, offering a solid foundation for understanding the demographic component of coastal vulnerability assessments.

## Results and discussion

### Physical coastal vulnerability index (PCVI)

#### Dawlish

##### Analysis of the PCVI values

**Fig. 10:**
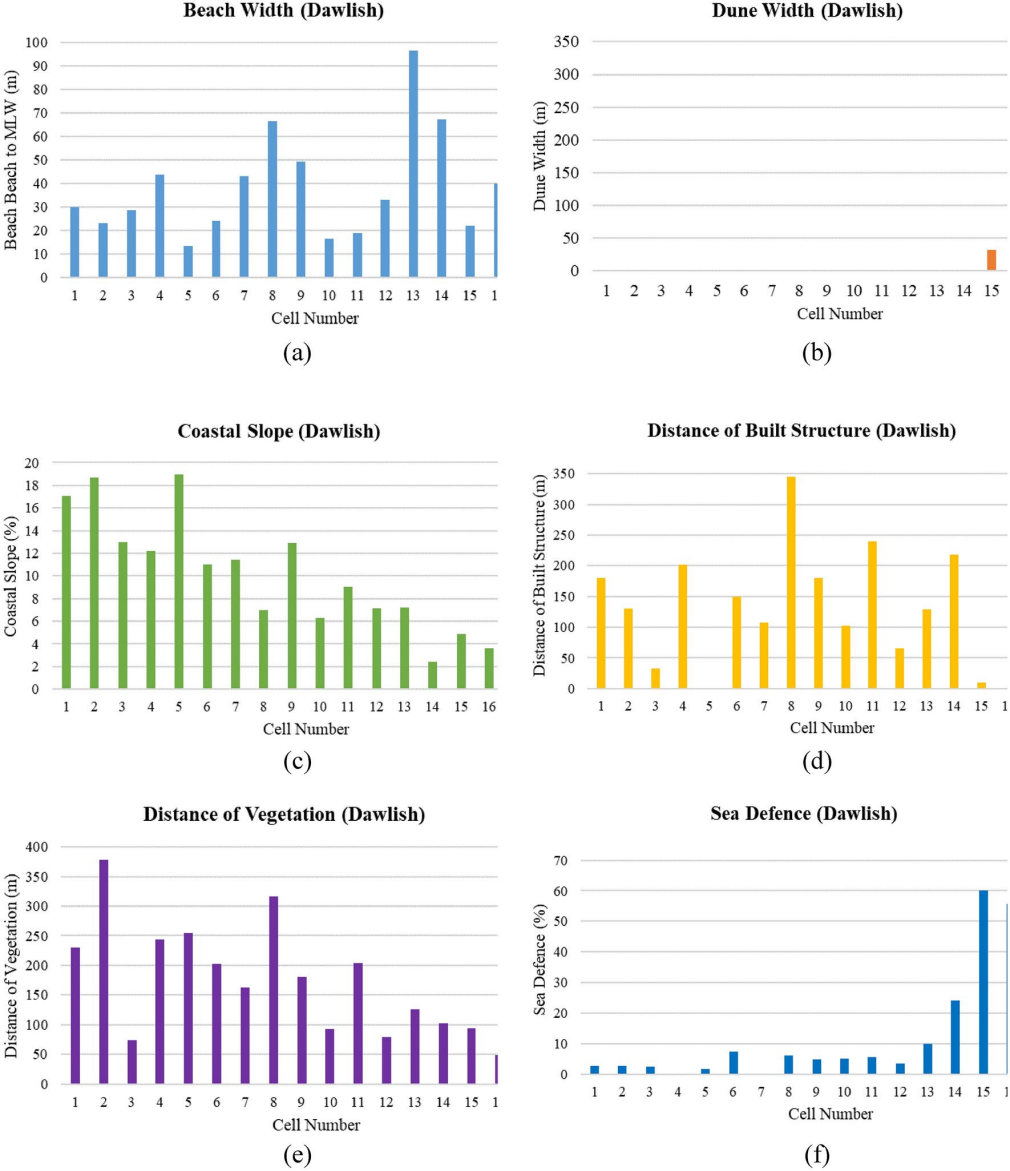
(**a**–**f**) Physical parameter measurement for Dawlish cells (Graphical Presentation).

In the 18 coastal cells of Dawlish, beach width varies significantly, measuring 37.5 m on average (Fig. 10a–f). The widest beach is found in cell 13 at 96.5 m, while the narrowest is in cell 5 at 13.2 m. Dune coverage is limited, observed in only 17% of the cells, with widths ranging from a minimal 16.3 m in cell 17 to a substantial 305 m in cell 18. Coastal slopes across the cells also vary significantly, from a gentle 2.4% in cell 14 to a steep 19% in cell 5, with an overall average slope of 9.7%. Regarding built structures, these are completely absent behind the back beach in 22% of the cells, while the total length of built structures spans up to 345.23 m in cell 8, indicating extensive human activity and development in certain areas. The average length of built structures behind the back beach across all cells is 130.06 m. Vegetation extends an average of 158.74 m behind the back beach, with the most extensive vegetation found in cell 2, covering 378.5 m, and the least in cell 18 at 19.53 m. Sea defences are installed in 61% of the cells, with coverage ranging from 69.42% in cell 18 to none in several others.

##### Overall PCVI scores and trend

**Fig. 11:**
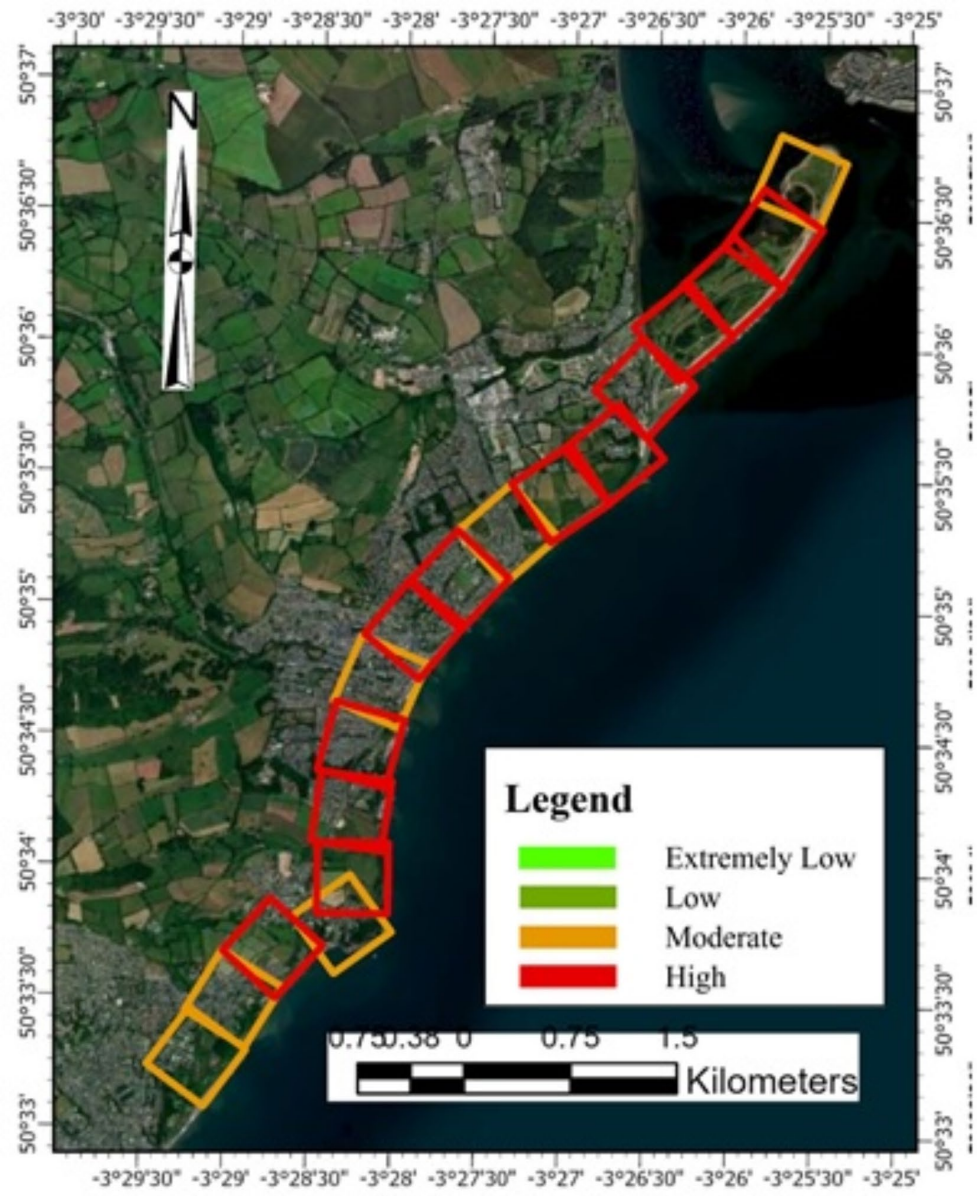
PCVI GIS vulnerability map for Dawlish. *Source* This figure was created by the second author using ArcGIS 10.3.1 version on the Google Pro maps.

The PCVI scores for Dawlish (Fig. 11) show varying degrees of vulnerability across the 18 assessed cells. The PCVI scores range from a low of 17 in cells 4 and 18, suggesting these areas may be less vulnerable to physical coastal threats, to a high of 23 in cell 12, indicating greater vulnerability to physical coastal processes such as erosion or flooding. Most cells have a PCVI score ranging from 18 to 21, denoting a moderate level of vulnerability, with cell 10 standing out with a score of 22, just one point lower than the highest recorded score in cell 12. These two cells can be considered the most vulnerable within the studied area and may require more focused attention in terms of management and protective measures. Conversely, cells with the lowest scores may indicate areas where existing coastal defences or natural features offer greater protection. According to the National Coastal Erosion Risk Mapping Project indicates, 42% of the coastline in England and Wales is at risk of erosion, with 82% of this coastline currently undefended^90^.

#### Aberystwyth

##### Analysis of the PCVI values

**Fig. 12:**
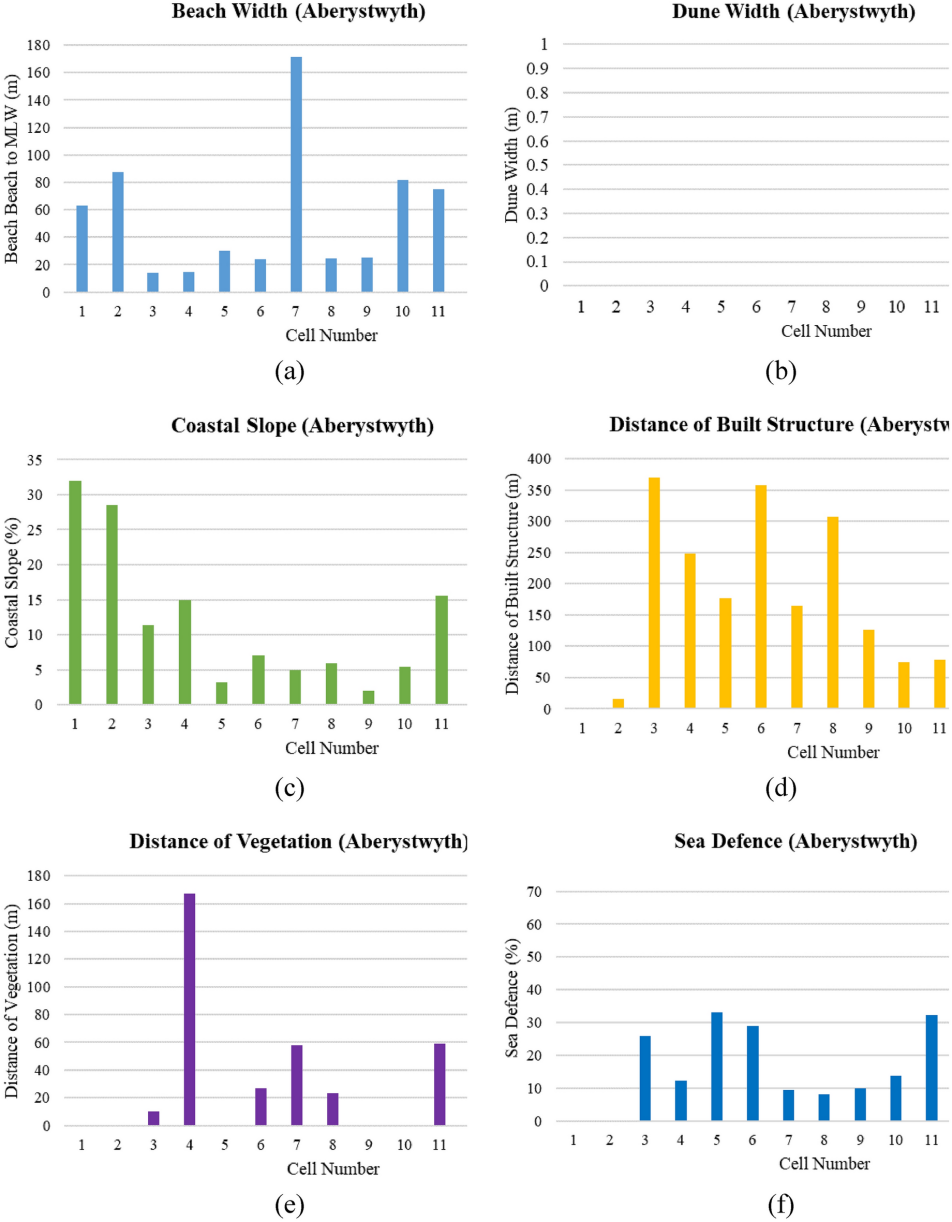
(**a**–**f**) Physical parameter measurement for Aberystwyth cells (Graphical Presentation).

In assessing coastal vulnerability for Aberystwyth, among 15 cells (Fig. 12a–f), the average beach width is 47.9 m, with cell 7 having the widest beach at 171.5 m, and cell 3 having the narrowest at 14.2 m. Dune width is consistently recorded at zero across all cells, indicating an absence of this natural protective feature throughout the region. Coastal slopes vary, with an average of 12.1%, suggesting different levels of exposure to wave action; cell 1 has the steepest slope at 32%, potentially increasing erosion rates, while cell 15 has the gentlest slope at 1.4%, which may allow for better wave energy dissipation. Built structures are absent in 40% of the cells, potentially increasing the vulnerability of these areas to coastal hazards due to the lack of physical barriers. The longest span of built structures, measuring 370.35 m, is found in cell 3, providing a significant man-made protective buffer. Vegetation is also notably scarce, with 73% of the cells having no vegetative cover behind the back beach, which could otherwise offer stabilization and natural protection against coastal processes. Sea defences are present in a minority of the cells, with the highest coverage at 33.08% in cell 5, reflecting efforts to reduce vulnerability through artificial means where natural structures are lacking. However, the absence of sea defences in many cells, highlights areas that may require additional attention in coastal vulnerability mitigation strategies. Together, these physical characteristics provide a comprehensive view of the region’s resilience against coastal threats, emphasizing the need for tailored management approaches in Aberystwyth to address the unique vulnerabilities of each cell.

##### Overall PCVI scores and trend

**Fig. 13:**
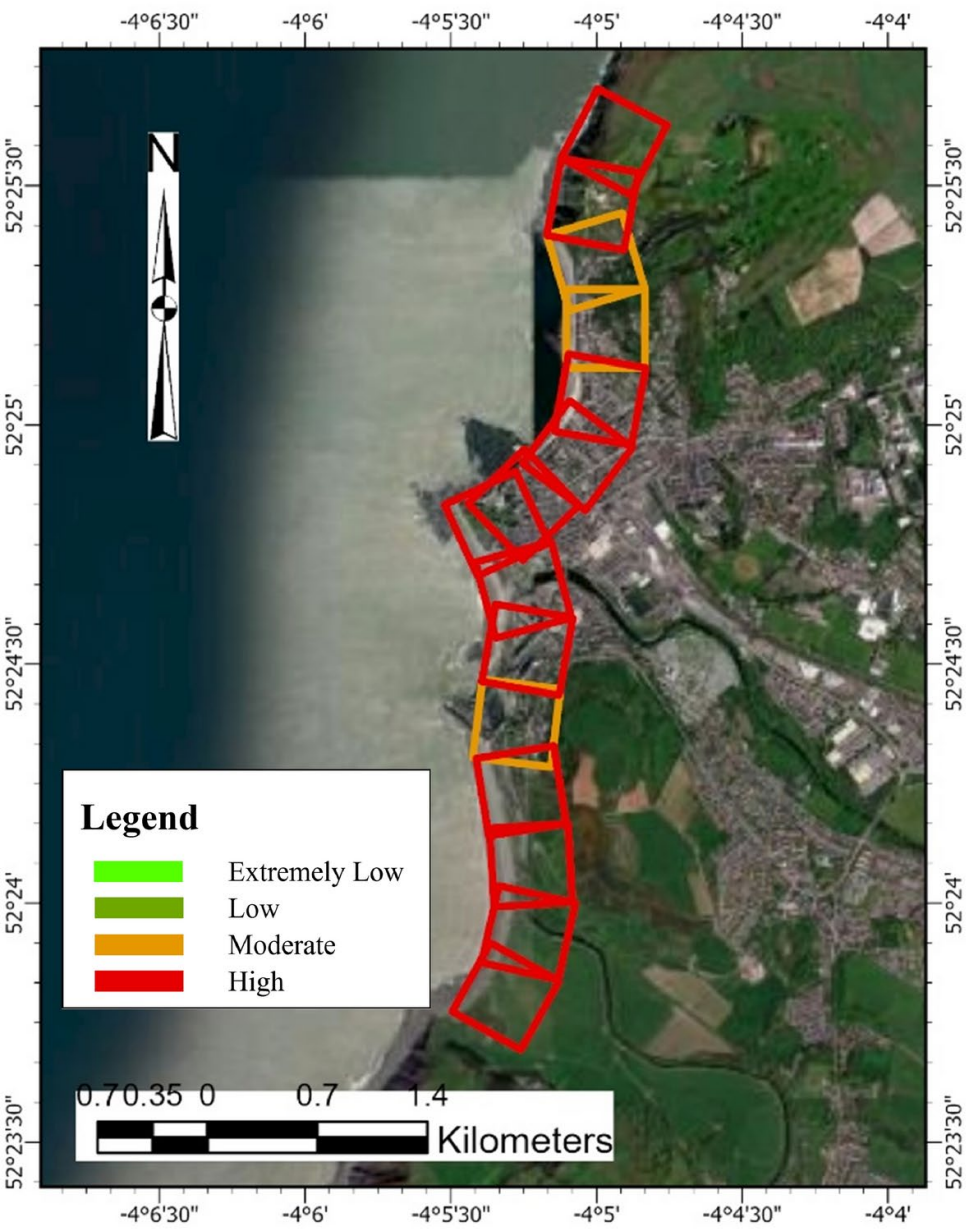
Physical vulnerability map for Aberystwyth. *Source* This figure was created by the second author using ArcGIS 10.3.1 version on the Google Pro maps.

The PCVI for Aberystwyth shows variability across the 15 cells (Fig. 13), indicating a range of vulnerability levels along this segment of the coastline. Cells 14 and 15, with the highest PCVI scores of 24, demonstrate the greatest vulnerability, which may reflect an increased risk from coastal hazards such as erosion or inundation. In contrast, cell 4, with the lowest score of 17, appears less vulnerable to physical coastal threats. Most cells have PCVI scores between 18 and 21, denoting a moderate level of vulnerability that still warrants attention in coastal management strategies. Cell 9, with a PCVI score of 22, stands out as having a slightly higher vulnerability than the median for the area. These scores are essential in informing prioritization for coastal defence measures, management strategies, and potential areas for conservation or restoration to enhance resilience.

#### Happisburgh

##### Analysis of the PCVI values

**Fig. 14:**
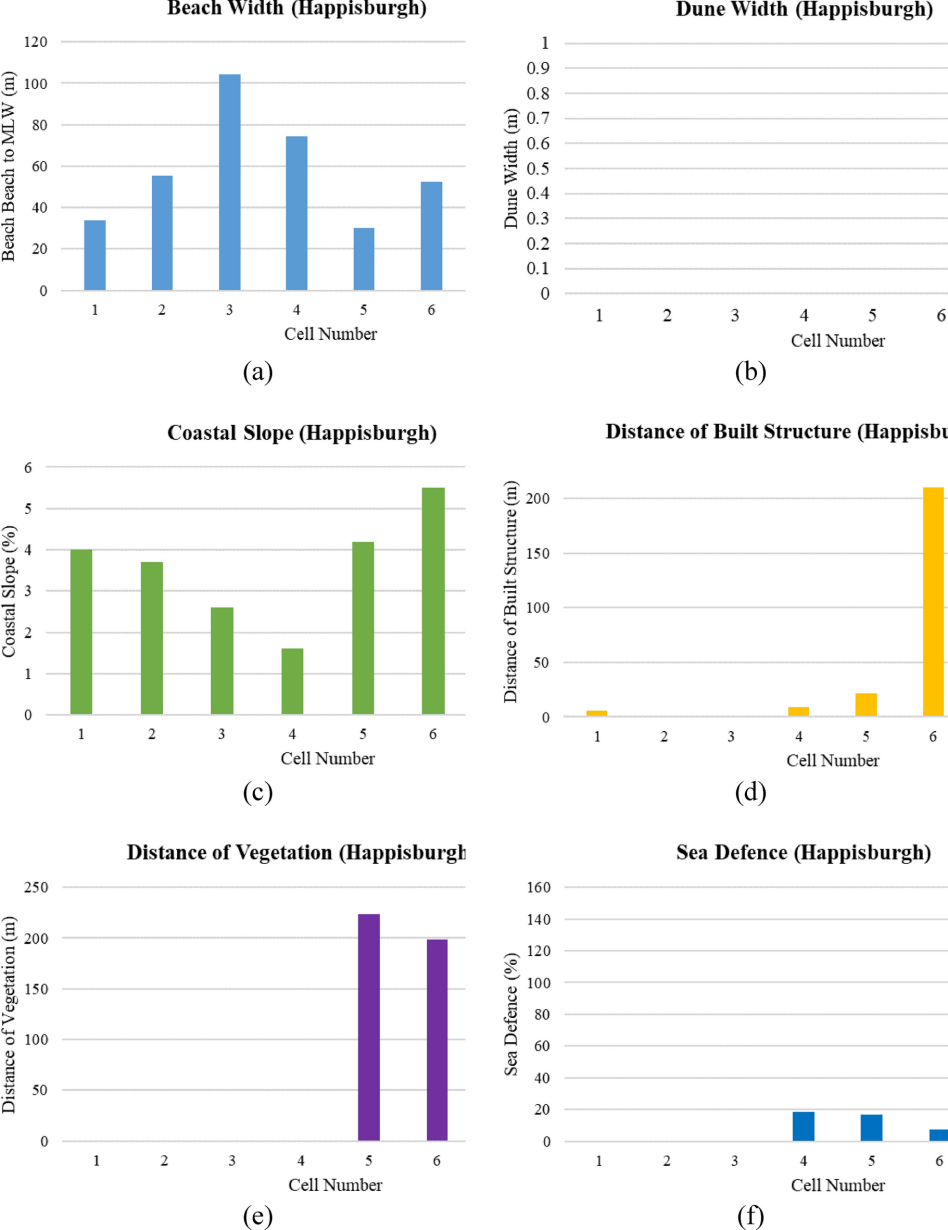
(**a**–**f**) Physical parameter measurement for Happisburgh cells (Graphical Presentation).

For Happisburgh, an analysis of the CVI based on physical parameters across 8 cells provides an insightful overview (Fig. 14a–f). The average distance to mean low water (MLW) across these cells is 48.6 m, indicating the available space for wave energy dissipation before impacting the shore. Cell 3 has the widest beach at 104.4 m, offering a significant buffer zone, while cell 7 has the smallest beach width at just 11.5 m, suggesting a higher exposure to coastal hazards. The average coastal slope across the cells is 4.9%, with a gentler of 1.6% in cell 4, which may help reduce wave energy intensity, and a steeper slope of 5.5% in cell 6, potentially increasing erosion rates. The total length of built structures behind the back beach varies, with a notable 210.71 m in cell 6, indicating a considerable human footprint that may influence coastal processes and management decisions. In contrast, 75% of the cells, including cells 2, 3, and 7, have no built structures, suggesting less direct human influence and possibly a greater reliance on natural coastal dynamics. Vegetation behind the back beach is significant in cell 5, covering 223.71 m, which can serve as a natural protective barrier and enhance the shoreline’s stability. In terms of sea defences, cell 8 stands out with a 155% level of intervention, indicating significant efforts to protect the coast in this area. Conversely, half of the cells have no recorded sea defences, which may suggest either a reliance on natural landforms for protection or a potential gap in the coastal defence infrastructure.

##### Overall PCVI scores and trend

**Fig. 15:**
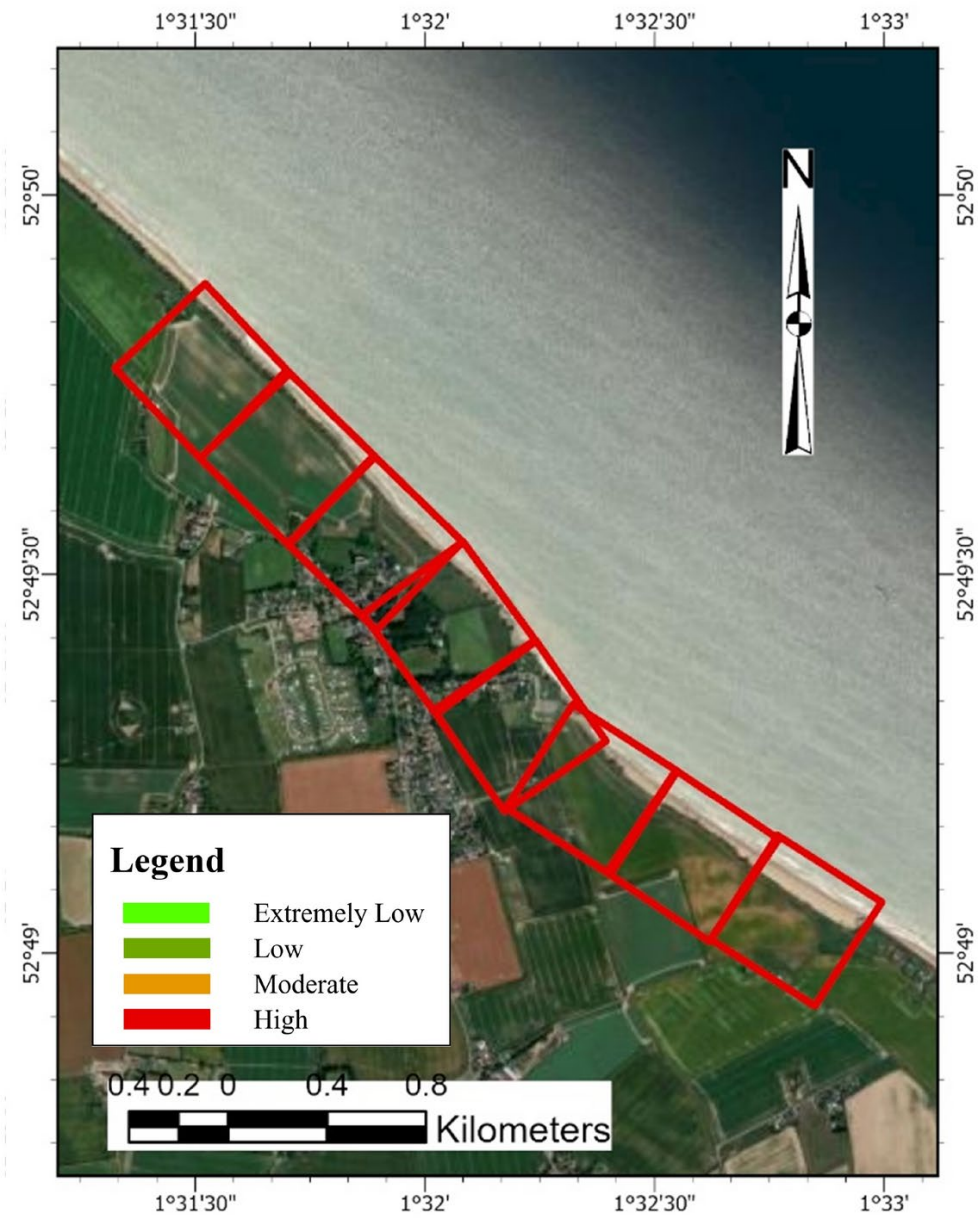
Physical vulnerability map of Happisburgh. *Source* This figure was created by the second author using ArcGIS 10.3.1 version on the Google Pro maps.

From Fig. 6, the PCVI for Happisburgh across 8 cells (Fig. 15), reveals a range of vulnerability levels. Cells 1, 2, and 7 share the highest PCVI score of 23, indicating a relatively high level of vulnerability to coastal hazards within the region. This high score suggests greater exposure to erosion, storm surges, or other coastal risks, necessitating careful management and potential reinforcement of coastal defences. Cells 3 and 4 each have a PCVI score of 22, indicating a slightly lower but still significant potential vulnerability compared to the highest-scoring cells. These areas may also require attention to effectively mitigate risks and protect against the impacts of coastal processes. Conversely, cells 5 and 8, with scores of 20, and cell 6, with the lowest score of 19, appear less vulnerable according to the PCVI assessment. These areas are less exposed to severe coastal dynamics based on geographic and physical characteristics.

#### Combined CVI scores and trends of PCVI

**Fig. 16:**
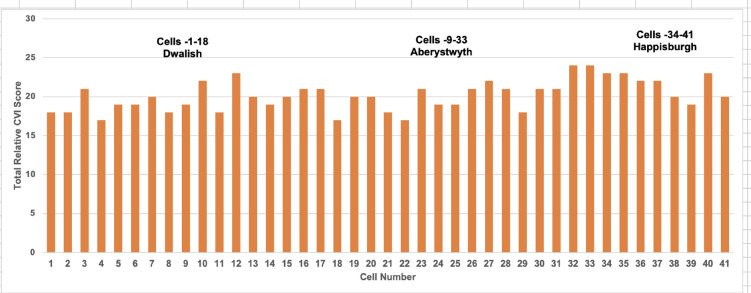
Cumulative scores of PCVI.

**Fig. 17:**
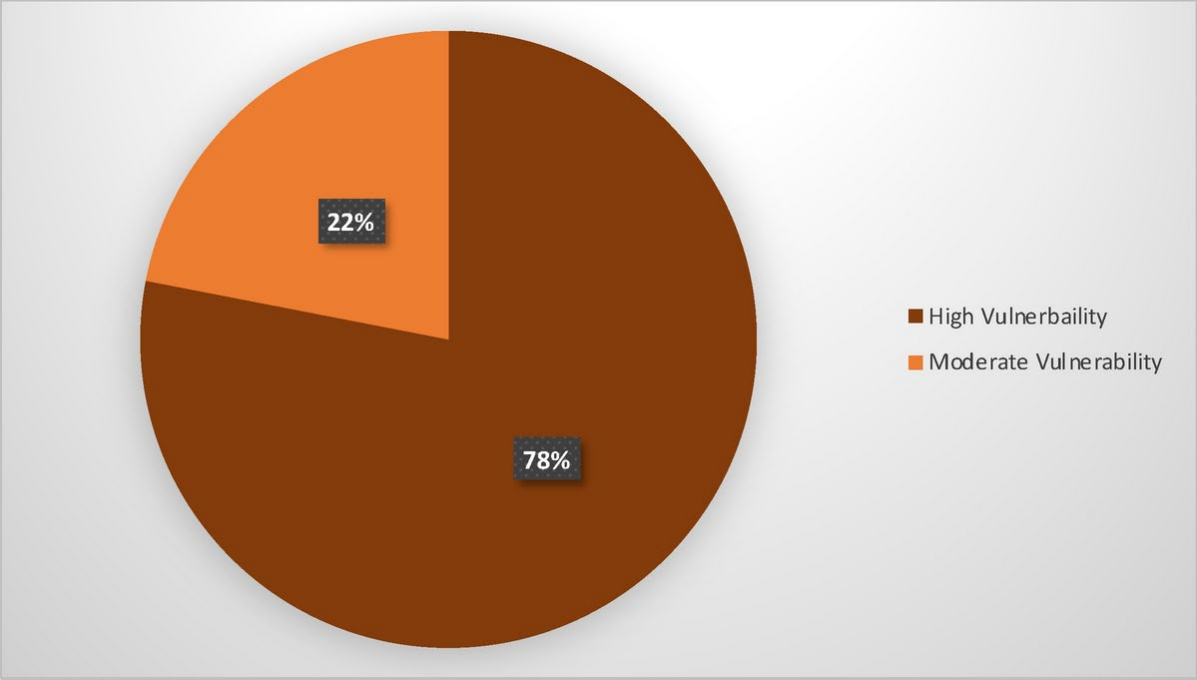
Cumulative scores of PCVI in percentage.

Based on the cumulative PCVI scores, nearly 78% of cells, or 32 out of 41 cells, are highly vulnerable (Figs. 16 and 17). More than 20% of cells, specifically 9, fall into the moderate vulnerability category. Consequently, this study concludes that all three regions are highly vulnerable. The highest vulnerability, with a score of 24, was observed in cells 14 and 15, located in Aberystwyth, while the second highest vulnerability, scoring 23, was recorded in cells 1, 2, and 7 of Happisburgh. The lowest vulnerability, with a score of 17, was recorded in cell 18 of Dawlish and cell 4 of Happisburgh. The PCVI trends indicate a high level of vulnerability across the studied cells.

Over the past century, beach steepening has been observed across England and Wales; however, it is less prevalent along the southeast coast of England^91^.

### Economic coastal vulnerability index (ECVI)

The detailed economic parameter measurements and their corresponding CVI ratings for Dawlish, Aberystwyth, and Happisburgh are included in Appendix C and Appendix D, respectively.

#### Dawlish

##### Analysis of the ECVI values

**Fig. 18:**
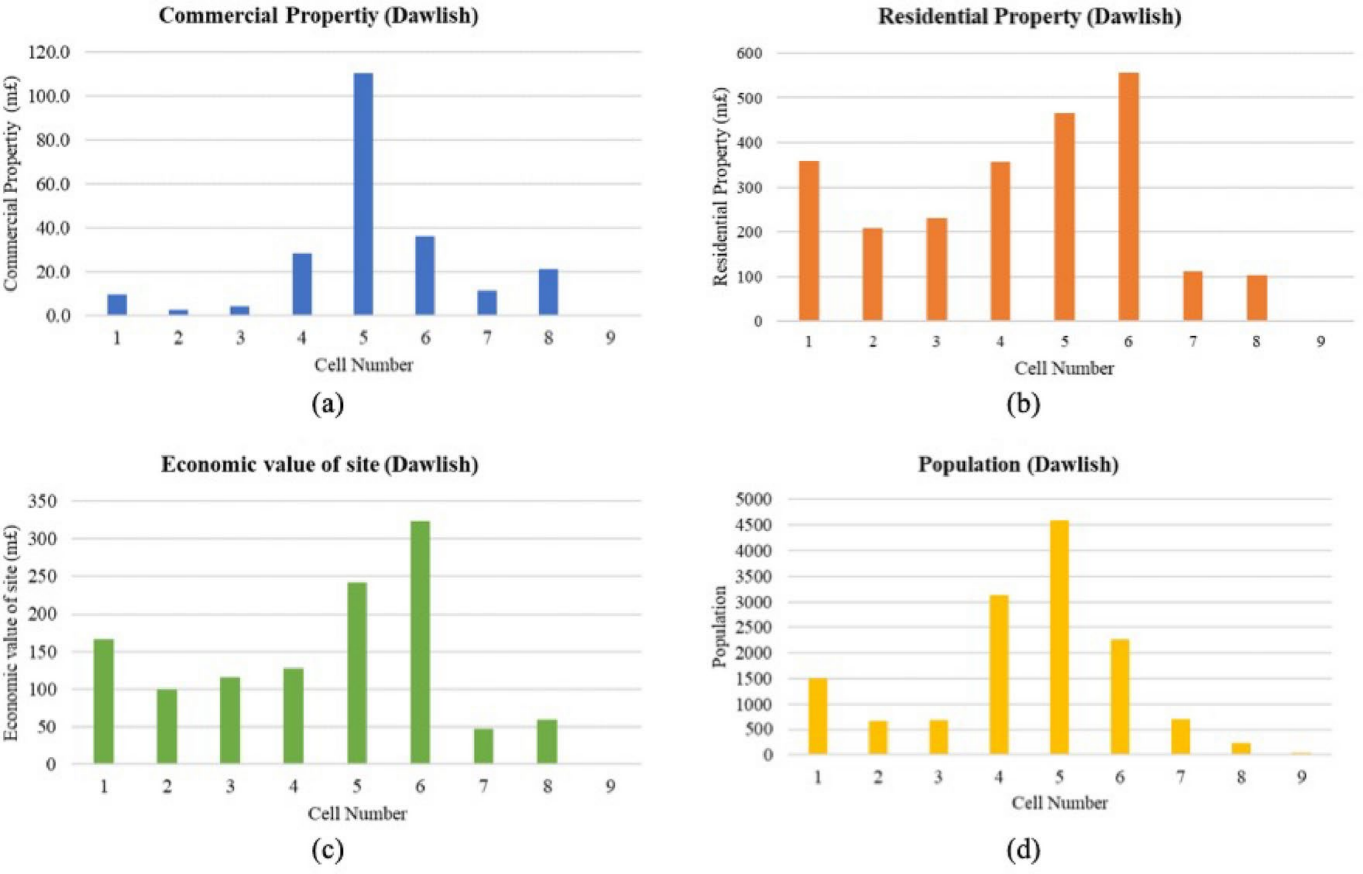
(**a**–**d**) Economic parameter measurement for Dawlish cells (Graphical Presentation).

In Dawlish, the economic attributes of coastal cells reveal a diverse economic landscape, impacting strategies for coastal vulnerability management (18a-d). Among 9 cells, cell 5 stands out as the economic powerhouse, with staggering commercial and residential property values exceeding £110 million and £465 million, respectively, and it has the highest population count of 4593. This denotes a dense concentration of assets and people at risk. Conversely, Cell 9 has no recorded commercial and residential value and supports a minimal population, possibly indicating undeveloped land or public spaces with different management requirements. Cells 4 and 6 also hold substantial economic stakes, with residential property values reaching over £356 million and £556 million, respectively, and hosting large populations that emphasize the need for robust coastal defence mechanisms. Cells 1, 2, and 3, while less economically valued than Cells 4, 5, and 6, still present significant economic figures and support hundreds of residents. These figures underscore the need for a tiered approach to coastal protection, prioritizing areas like Cell 5 due to its high economic and demographic significance while also addressing the need for cells with lower, yet substantial, economic valuations (Fig. 18).

##### Overall ECVI scores and trend

**Fig. 19:**
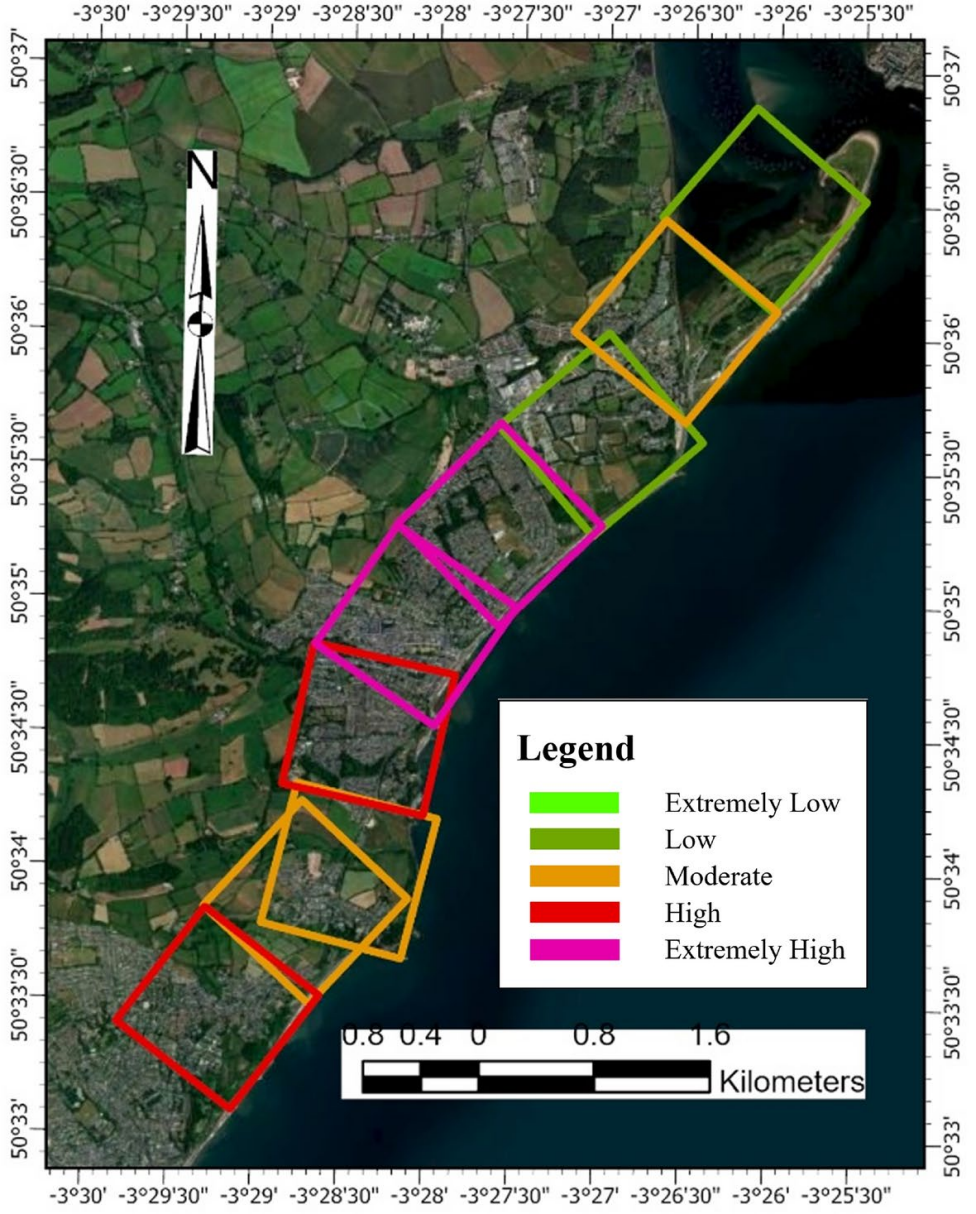
Economic vulnerability map for Dawlish. *Source* This figure was created by the second author using ArcGIS 10.3.1 Version on the Google Pro maps.

In Dawlish, the ECVI (Fig. 19) varies significantly, with scores ranging from 4 to 18, reflecting diverse economic risks associated with coastal vulnerability. Cells 5 and 6 exhibit the highest ECVI scores of 18 and 17, respectively. These scores are attributed to their top rankings in both commercial and residential property values, as well as the economic value of the site, combined with a high population score. This indicates a significant concentration of economic assets and population density, heightening the potential impact of coastal events on these cells. Cells 1, 2, and 3 show moderate ECVI scores ranging from 11 to 13, despite having the highest possible scores for residential property and economic value of the site. This reflects a disparity between economic valuation and population density, suggesting that while these areas may be economically significant, they are less densely populated and may face a moderately different set of vulnerabilities. On the lower end of the ECVI spectrum, Cell 9 scores the lowest at 4, with the lowest rankings across all economic parameters, indicating a relatively low economic exposure to coastal hazards. Cells 7 and 8 have low to moderate ECVI scores of 8 and 10, respectively, with lower commercial and residential property values and lower economic site values, denoting lesser economic vulnerability than other cells.

#### Aberystwyth

##### Analysis of the ECVI values

**Fig. 20:**
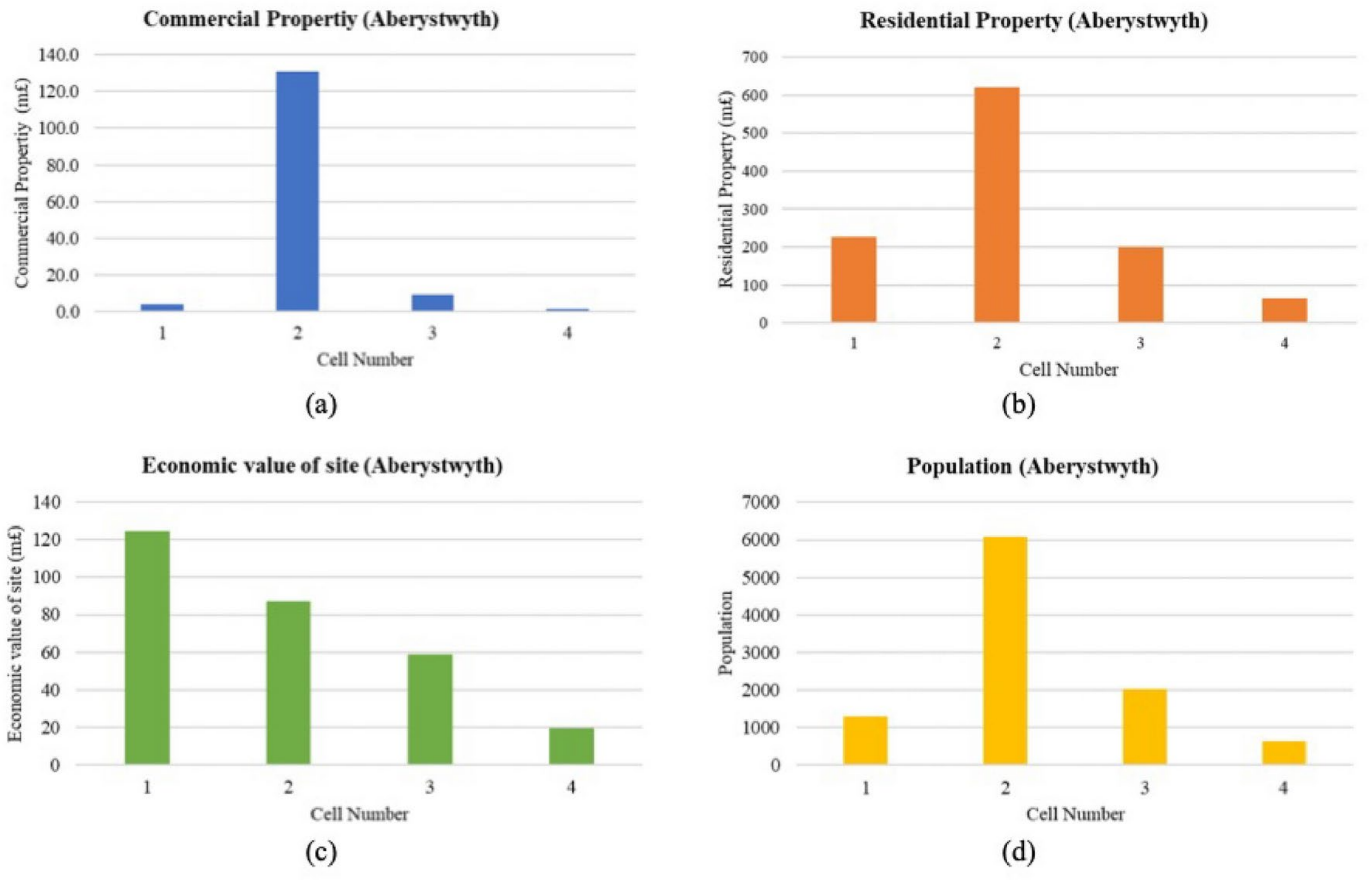
(**a**–**d**) Economic parameters measurement for Aberystwyth cells (Graphical Presentation).

In Aberystwyth, the distribution of economic parameters across 4 cells highlights variation in economic and demographic factors that contribute to the area’s coastal vulnerability profile (Fig. 20a–d). Cell 2 stands out with the highest commercial property value at over £130 million and the highest residential property value at over £619 million, accompanied by the largest population of 6079. This high concentration of economic assets and population density suggests that Cell 2 is a critical area for investment in coastal protection measures. Cell 1, although it has a lower commercial property value around £4 million, still holds a substantial residential property value at over £227 million and supports a population of 1287. The economic value of the site is notable, exceeding £124 million, indicating significant investment and potential risk from coastal threats. Cell 3 has commercial and residential values of approximately £9 million and £199 million, respectively, with a population of 2026, reflecting a moderate level of development and economic significance. Cell 4 is the least developed in terms of economic metrics, with the lowest commercial and residential property values, at approximately £1.3 million and £65 million, respectively, and the smallest population of 644. Its economic site value of around £19 million suggests it may have lower exposure to economic loss due to coastal hazards.

##### Overall ECVI scores and trend

**Fig. 21:**
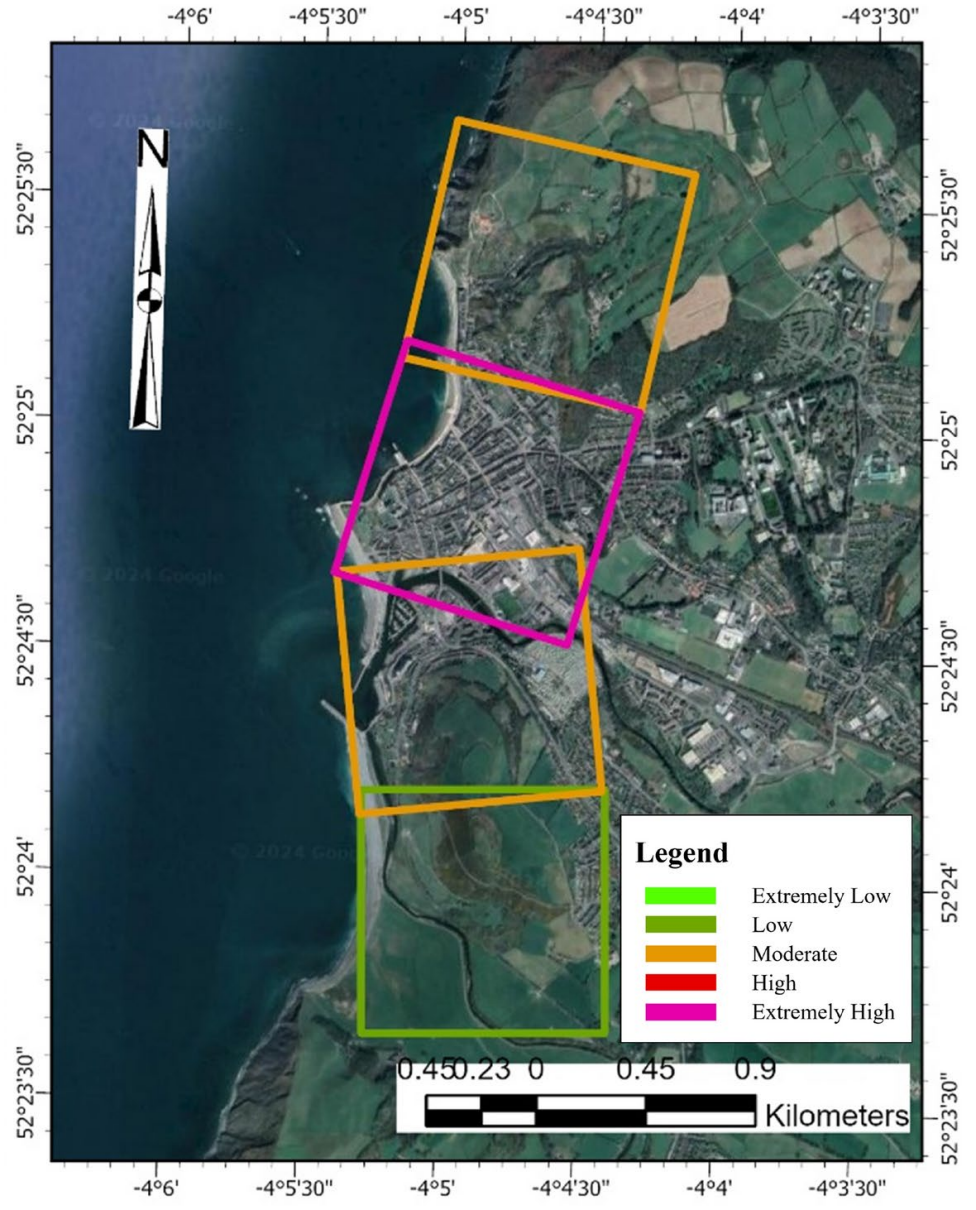
Economic vulnerability map for Aberystwyth. *Source* This figure was created by the second author using ArcGIS 10.3.1 version on the Google Pro maps.

Cell 2, with an ECVI score of 17, exhibits the highest potential economic risk (Fig. 21), correlating with its high commercial and residential property values and a large population. This indicates a dense hub of economic and social activity that could be significantly impacted by coastal events. Cells 1 and 3, both with an ECVI of 12, represent moderate economic vulnerability, despite having the highest scores for residential property. This suggests that the residential areas in these cells are of high value, with Cell 1 also possessing a substantial economic value for its site. The presence of larger populations in these areas highlights the urgency for protective measures to mitigate potential economic losses from coastal hazards. Cell 4, with the lowest ECVI score of 7, reflects the least economic vulnerability among the assessed cells. Its lower scores in residential property and economic value of the site, paired with a smaller population, indicate a reduced economic risk from coastal threats, potentially allowing for more flexible management options.

#### Happisburgh

##### Analysis of the ECVI values

**Fig. 22:**
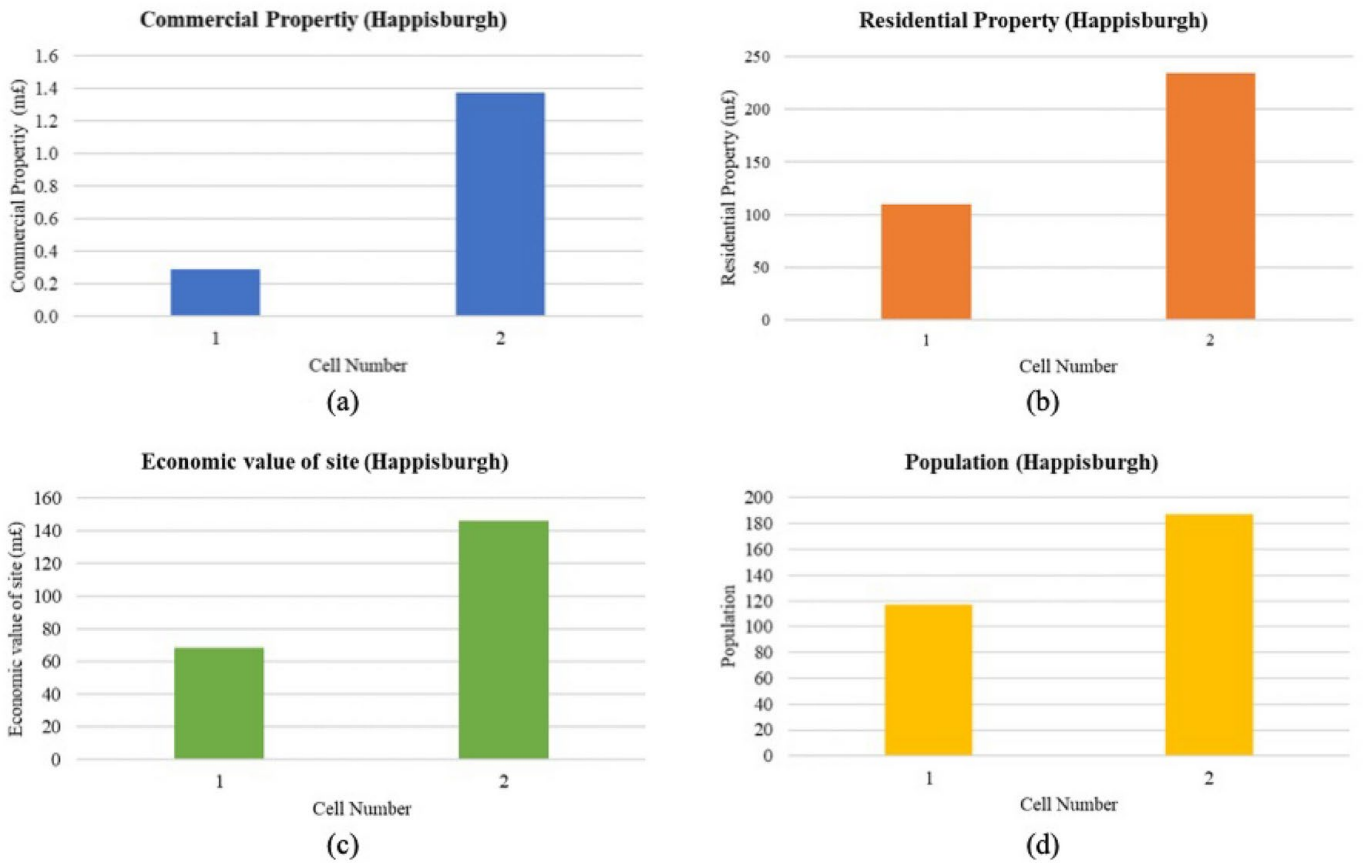
(**a**–**d**) Economic parameter measurement for Happisburgh cells (Graphical Presentation.

Cell 1, with commercial property valued at £289,995 and residential property at over £110 million, has an economic site value of approximately £68.5 million. Despite supporting a smaller population of 117, the economic stakes in terms of property values are considerable. In contrast, Cell 2 shows a higher level of economic investment, with commercial property valued at over £1.3 million and residential property exceeding £234 million. The economic value of this site is notably higher at around £146 million, and with a larger population of 187, Cell 2 stands out as a significant economic and social hub within Happisburgh (Fig. 22a–d). The high economic values in this cell suggest that it may require prioritized attention for coastal defence and resilience planning.

##### Overall ECVI scores and trend

**Fig. 23:**
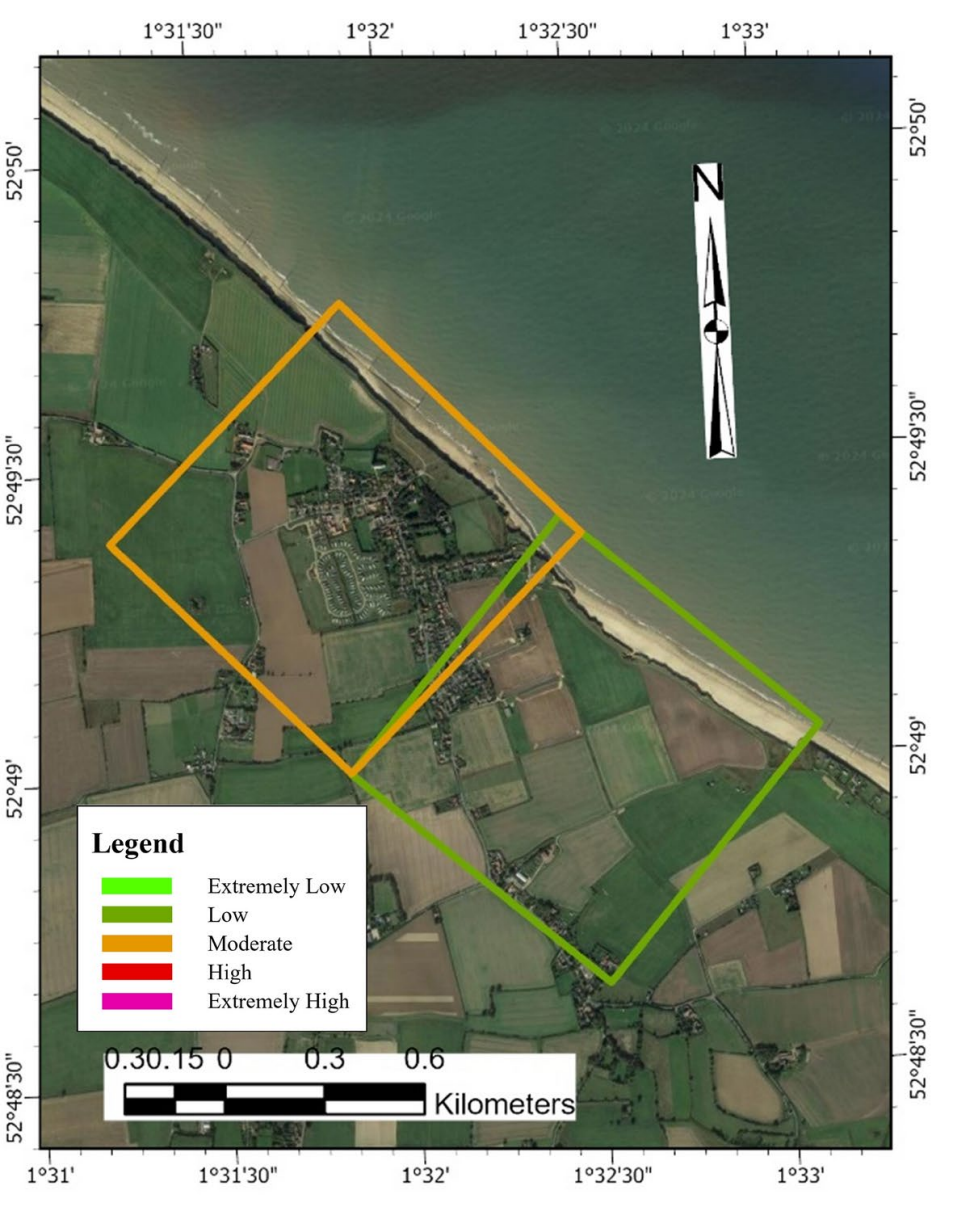
Economic vulnerability map for Happisburgh. *Source* This figure was created by the second author using ArcGIS 10.3.1 version on the Google Pro maps.

For Happisburgh, the ECVI (Fig. 23) indicates a differential in economic exposure to coastal threats between the two cells. Cell 2, with an ECVI score of 11, shows a higher economic vulnerability, which is reflected by the highest scores for residential property and economic value of the site within the region. This score, coupled with a consistent population score, emphasizes a concentration of assets that could be at risk from coastal hazards. In contrast, Cell 1 has a lower ECVI score of 8, characterized by moderate residential property and economic site values. Despite sharing the same commercial property and population scores as Cell 2, the lower residential and economic values in Cell 1 suggest a comparatively reduced potential for economic loss due to coastal threats.

#### Cumulative ECVI scores and trends

**Fig. 24:**
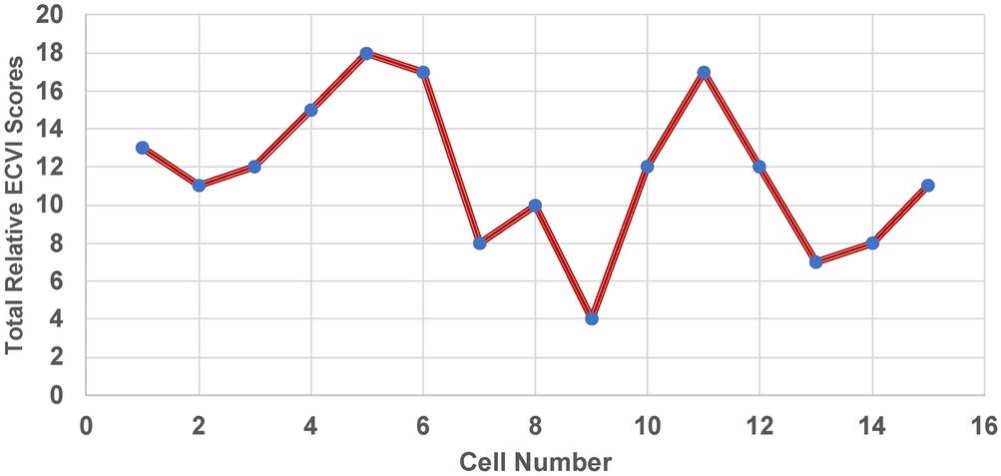
Cumulative CVI scores of ECVI.

**Fig. 25:**
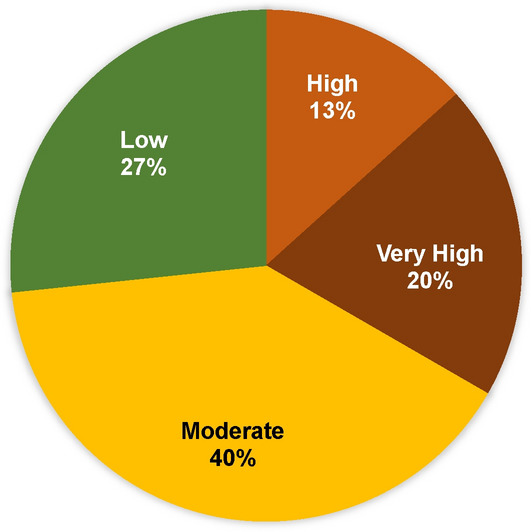
Percentage of cumulative CVI scores of ECVI.

The ECVI scores show different results when compared to the PCVI scores. Based on the aggregated ECVI scores, approximately 33% of cells are classified as high to very highly vulnerable (Figs. 24 and 25), which corresponds to 5 out of 15 cells. Nearly 40% of cells, specifically 5 cells, fall into the moderate vulnerability category. The highest vulnerability, with a score of 18, was observed in Cell 5, located in Dawlish, while the second highest vulnerability, with a score of 17, was recorded in Cell 6 of Dawlish and Cell 2 of Aberystwyth. The lowest vulnerability, with a score of 4, was recorded in Cell 9 of Dawlish, and the second lowest was recorded in Cell 7 of the same site. The ECVI trends indicate a range from moderate to very high levels of vulnerability.

### Combined coastal vulnerability

The calculation of the CCVI involved aggregating scores from both PCVI and ECVI as outlined in Table 6. For example, in Dawlish, the PCVI score across 18 cells summed to 350, resulting in an average of 19.4. Additionally, Dawlish’s ECVI score across 9 cells totalled 108, with an average of 12. Dawlish’s CCVI was then calculated by using Eq. (3) as follows.

**Table 6:** CCVI for Dawlish, Aberystwyth, and Happisburgh.

Region	Average PCVI	Average ECVI	CCVI
Dawlish	19.44	12.00	15.7
Aberystwyth	20.40	12.00	16.2
Happisburgh	21.50	9.50	15.5

$$CCVI=\frac{19.4 +12}{2}=15.7$$

**Fig. 26:**
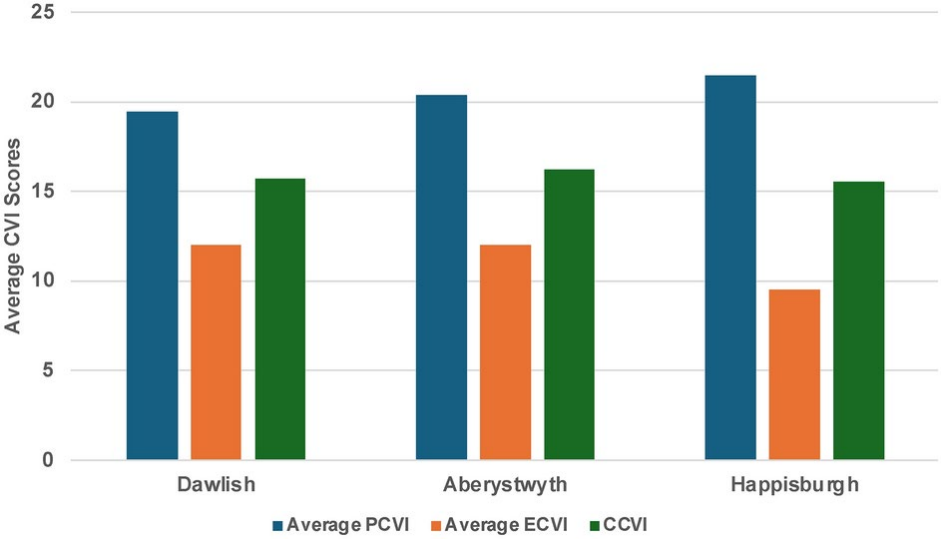
Combined coastal vulnerability for Dawlish, Aberystwyth, and Happisburgh.

Dawlish and Aberystwyth have the same average ECVI of 12, indicating potential economic impacts from coastal threats, despite Dawlish having a slightly lower PCVI (Table 6 and Fig. 26). Aberystwyth, with the highest CCVI at 16.2, suggests it may face the most considerable overall vulnerability. Happisburgh, while having the highest PCVI, indicates greater physical exposure but has the lowest ECVI, suggesting less economic risk. Its CCVI of 15.5, close to Dawlish’s 15.7, points to a similar level of overall vulnerability despite differing physical and economic risk factors.

## Correlation of parameters

### Physical parameters

Table 7 presents results on how physical features such as beach width, coastal slope, and distances to Mean Low Water (MLW) interact with sea defences and other coastal management factors in each region. A strong positive correlation (r = 0.6176, *p* = 0.0063) in Dawlish indicates that wider dunes tend to have more substantial sea defences, reflecting their pivotal role in coastal management. In Dawlish, coastal management efforts focus on areas with wide dunes and flatter slopes, providing additional sea defences. Steeper slopes naturally offer more protection^92^, reducing the need for extensive human interventions. A significant positive correlation shows that steeper coastal slopes are associated with larger distances to MLW, indicating natural protection or erosion processes at play. Additionally, a significant negative correlation between sea defence and coastal slope suggests that areas with steeper slopes may require fewer man-made sea defences, possibly due to natural topographical protection. This region shows a clear interaction between the physical landscape and human interventions.

**Table 7:** Correlation of physical parameters.

		Beach width to MLW	Dune	Coastal slope	Distance of built structure	Distance of vegetation	Sea defence
Combined	Beach width to MLW	1					
	Dune width	− 0.0743 (0.6441)	1				
	Coastal slope	− 0.0274 (0.8647)	− 0.1372 (0.3923)	1			
	Distance of built structure	− 0.0648 (0.6872)	− 0.1846 (0.248)	0.0111 (0.945)	1		
	Distance of vegetation	− 0.1579 (0.3240)	− 0.1155 (0.4721)	0.2533 (0.1101)	0.3751* (0.0157)	1	
	Sea defence	− 0.0901 (0.5753)	0.3496* (0.0251)	− 0.2768 (0.0798)	− 0.1495 (0.3510)	− 0.2123 (0.1826)	1
Dawlish	Beach width to MLW	1					
	Dune width	− 0.0687 (0.7866)	1				
	Coastal slope	− 0.3256 (0.1873)	− 0.3259 (0.1869)	1			
	Distance of built structure	0.414 (0.0877)	− 0.3721 (0.1283)	0.1076 (0.6709)	1		
	Distance of vegetation	− 0.0398 (0.8755)	− 0.4231 (0.0802)	0.7078* (0.0010)	0.5955* (0.0091)	1	
	Sea defence	− 0.0549 (0.8287)	0.6176* (0.0063	− 0.6703* (0.0023)	− 0.5506* (0.0179)	− 0.6038* (0.0080)	1
Aberystwyth	Beach width to MLW	1					
	Dune width	–	–				
	Coastal slope	0.1193 (0.6719)	–	1			
	Distance of built structure	− 0.2696 (0.3312)	–	− 0.2697 (0.3310)	1		
	Distance of vegetation	0.0285 (0.9197)	–	0.0762 (0.7873)	0.3614 (0.1856)	1	
	Sea defence	− 0.113 (0.6885)	–	− 0.2475 (0.3737)	0.6387* (0.0104)	0.2052 (0.4632)	1
Happisburgh	Beach width to MLW	1					
	Dune width	–	–				
	Coastal slope	− 0.4813 (0.2272)	–	1			
	Distance of built structure	0.0086 (0.9839)	–	0.5527 (0.1554)	1		
	Distance of vegetation	− 0.2071 (0.6227)	–	0.5104 (0.1963)	0.6721 (0.0679)	1	
	Sea defence	− 0.1675 (0.6917)	–	0.3935 (0.3348)	− 0.1623 (0.7010)	− 0.159 (0.7069)	1

Values within parentheses represent the statistical significance (*p*) values, which should be less than 0.05. A star on the correlation (r) indicates that the relationship is statistically significant.

In Aberystwyth, a weak, insignificant correlation between beach to MLW and the coastal slope (r = 0.1193, *p* = 6719), indicates a minimal direct relationship between these two variables. Other relationships, such as between beach width and coastal slope, also show weak, insignificant correlations, implying limited influence on each other. For Aberystwyth, the correlation analysis shows that none of the variables have a strong or statistically significant impact, highlighting that the beach and these coastal features are somewhat independent of each other in this region.

In Happisburgh, distance metrics tend to increase together, reflecting a coherent erosion or shoreline movement pattern, but lacking strong statistical relationships between physical features and sea defences. Sea defence and coastal slope correlate positively, but a weak correlation (r = 0.3935, *p* = 0.3348) shows a tendency for more sea defences in steeper areas, though the relationship is not statistically significant. Coastal slope and beach width correlate negatively and indicate that steeper slopes are linked to narrower beaches.

### Economic parameters

Table 8, which examines the correlation between economic parameters in all three study areas, suggests that commercial and residential activities are strongly interlinked with economic value and are major drivers of population growth. In both Dawlish and Happisburgh, residential activity is strongly correlated with economic value (*p* < 0.05), indicating that increased residential development boosts local economies. However, in Aberystwyth, this relationship is weaker, suggesting that economic value may depend on other factors in this region. In Happisburgh, perfect correlations are observed between commercial activity, residential activity, and economic value in the area. Generally, commercial activity, residential activity, and population are all strongly and significantly correlated, boosting the overall economic values in the study areas.

**Table 8:** Correlation of economic parameters.

		Commercial property	Residential property	Economic value of site	Population
Combined	Commercial property	1			
	Residential property	0.7671* (0.0008)	1		
	Economic value of site	0.3587 (0.1892)	0.7514* (0.0012)	1	
	Population	0.9257* (0)	0.8461* (0.0001)	0.3942 (0.1460)	1
Dawlish	Commercial property	1			
	Residential property	0.609 (0.0817)	1		
	Economic value of site	0.608 (0.0824)	0.9712* (0)	1	
	Population	0.8756* (0.0020)	0.8000* (0.0096)	0.7113* (0.0317)	1
Aberystwyth	Commercial property	1			
	Residential property	0.9635* (0.0365)	1		
	Economic value of site	0.2267 (0.7733)	0.4607 (0.5393)	1	
	Population	0.9833* (0.0167)	0.9803* (0.0197)	0.2859 (0.7141)	1
Happisburgh	Commercial property	1			
	Residential property	1.0000* (0)	1		
	Economic value of site	1.0000* (0)	1.0000* (0)	1	
	Population	1.0000* (0)	1 (1)	1.0000* (0)	1

Values within parentheses represent the statistical significance (*p*) values, which should be less than 0.05. A star on the correlation (r) indicates that the relationship is statistically significant.

## Comparison of current study results with global CVI results

Coastal vulnerability is evaluated at different levels worldwide, ranging from global to village levels. Numerous studies focus either on physical^57,93–98^ or socio-economic vulnerability^45,82,96,99–102^, yet there is limited research that considers both aspects when assessing coastal vulnerability. Consequently, the results of this research are compared with existing studies that combine both aspects of coastal vulnerability (Table 9). Table 9 outlines coastal vulnerability methodologies used in ten countries, evaluating different coastal zones worldwide by integrating both physical and socio-economic variables. The most common variable in the Physical Coastal Vulnerability Index is the coastal slope, and in socio-economic parameters, it is the population; these parameters are used to evaluate UK coastal vulnerability in this study. All these studies have generated GIS maps to rank coastal areas in various aspects. Although existing literature on combined physical and socio-economic coastal studies is limited, some researchers have attempted to develop CVI indices in several directions, as detailed in Table 9.

**Table 9:** Combined physical and socio-economic coastal vulnerability studies and used parameters: comparison of global studies with the current study.

Existing combined coastal vulnerability studies	Location	Brief explanation	Parameters used which are similar to our study
Integrated coastal vulnerability assessment: A methodology for coastal cities management integrating socioeconomic, physical and environmental dimensions—Case study of Região dos Lagos, Rio de Janeiro, Brazil^103^	Região dos Lagos, Brazil	This paper evaluated the integrated physical, socioeconomic and ecosystem dimensions of coastal vulnerability at the regional scale by using different physical and socio-economic parameters.	Back beach features, dunes, coastal elevation and population
Vulnerability assessment of coastal areas to sea level rise from the physical and socioeconomic parameters: case of the Gulf Coast of Bejaia, Algeria^104^	Gulf Coast of Bejaia, Algeria	This paper assessed the coastal vulnerability in both physical and socio-economic aspects by using both physical and socio-economic aspects.	Coastal slope, and population
Coastal vulnerability assessment: a case study of Samut Sakhon coastal zone^96^	Samut Sakhon, Thailand	This study used four physical variables and four socio-economic variables to assess the coastal vulnerability of the Samut Sakhon coastal zone.	Coastal slope, and population density
Erosion Hazard Vulnerability of US Coastal Counties^105^	United States	This study examined the coastal vulnerability of US coastal counties by combining a socioeconomic and physical variable.	Coastal slope, and population
How to Define Priorities in Coastal Vulnerability Assessment^106^	Apulian region city of Barletta, Italy	This study considered both physical and socio-economic variables.	Coastal elevation/slope, and population
Assessing coastal vulnerability: Development of a combined physical and economic index^5^	Selected regions in the United Kingdom	This study evaluated the coastal vulnerability of 11 coastal areas in the UK by using both physical and economic parameters in 2018.	Beach width, dune width, coastal slope, distance of vegetation, a distance of built structures behind the back beach, rocky outcrop, sea defences, commercial properties, residential properties, the economic value of the site, population, coastal erosion, and flood (event) impact
Development of an Integrated Coastal Vulnerability Index for the Ivorian Coast in West Africa^50^	Ivorian Coast West Africa	This study assessed the coastal vulnerability of the Ivorian coast by using both physical and socio-economic variables.	Coastal slope, and population
Assessment of the integrated coastal vulnerability index of Ghana toward future coastal infrastructure investment plans^63^	Ghana	This study evaluated the coastal vulnerability of Ghana by using both physical and social variables.	Coastal slope, and population
A multi-hazards coastal vulnerability index of the east coast of Peninsular Malaysia^49^	Malaysia	This study evaluated the coastal vulnerability of Peninsular Malaysia by considering physical and socio-economic variables.	Vegetation, coastal slope, and population density
Coastal vulnerability to climate change in China’s Bohai Economic Rim^97^	Coast of Bohai Economic Rim, China	This research study evaluated the Bohai Economic Rim coastal vulnerability by using physical, and socioeconomic variables.	Population
Vulnerability Assessment of English and Welsh Coastal Areas (Current Study)	United Kingdom	This study assessed UK coastal regions (three selected case studies) by using physical and economic parameters.	Beach width, dune width, coastal slope, distance of vegetation, a distance of built structures behind the back beach, sea defences, commercial properties, residential properties, the economic value of the site, and population

Table 9 provides a systematic comparison of our study results with those from global studies. It’s important to note that our study differs from the research by Kantamaneni et al.^5^ in terms of the parameters used. Kantamaneni et al. used 13 parameters, including both physical and economic factors, and focused on 11 coastal areas—six in England, three in Wales, and one in Scotland. In contrast, our study used 10 parameters, excluding rocky outcrops, coastal erosion, and flood event impact. We evaluated three areas, two in England and one in Wales. When compared to the results of Kantamaneni et al.^5^, our study shows higher physical vulnerability for Happisburgh and lower economic vulnerability, likely due to the fewer parameters included in the ECVI. These results also suggest that physical vulnerability is increasing year by year.

Since 1990, several researchers^70,72,107–109^ have introduced new parameters for assessing area suitability without geographical limitations. These parameters do not necessarily include standard measurements such as relative sea level change, mean significant wave height, and mean tide range. Today, data on sea level rise, tides, and wave heights are publicly available in many countries for both current and future scenarios up to the year 2100. In our research, we have incorporated a diverse set of widely accepted parameters to conduct a combined physical and economic vulnerability assessment. The methodology we used in this study can be easily applied to any suitable area without geographical limitations. While the current research results are highly feasible for implementation, the process may still require some time.

## Recommendations for further research

Future research can enhance the CCVI by incorporating new parameters or refining the measurement methods of existing ones to create a more accurate index that better reflects local conditions, providing a more comprehensive reference for policymakers. For example, the current measurement of PCVI parameters is based on a single baseline, which is one-dimensional and may not fully capture the complexity of conditions within a cell. However, the results have been compared with similar studies and validated accordingly. In future studies, two-dimensional or three-dimensional datasets could be utilized, and spatial analysis through ArcGIS Pro could be employed to analyse multi-dimensional data, such as the coverage of vegetation and buildings, for a more thorough assessment of vulnerability. Additionally, this study was unable to include certain parameters, such as shoreline change, relative sea level change, mean significant wave height, and mean tide range, as physical parameters due to specific limitations. However, the current parameters reflect several factors, including structural, geomorphological, land use, land cover, and locational characteristics. To address these limitations, we utilized existing physical and economic parameters that capture these characteristics. In future research, we aim to include additional parameters, conduct correlation and sensitivity analyses, and construct more comprehensive indices. This will improve the coastal vulnerability analysis, making it more informative and valuable for policymakers.

## Conclusions

The current study utilized the CVI parameter-based method to provide updated insights into three selected coastal case study areas in the United Kingdom. This method combined physical (PCVI) and economic (ECVI) parameters to develop a comprehensive methodology (CCVI) for effective assessment at regional and sub-regional scales. The CCVI indices were applied to two regions in England (Dawlish, Happisburgh) and one in Wales (Aberystwyth). The scale of measurement and data sources played a crucial role in adapting this approach to the three distinct geographical locations. Dawlish, a region in England with significant economic importance, requires focused protective measures. In contrast, Happisburgh exhibits the greatest physical vulnerability, while Aberystwyth is identified as the most at-risk area in Wales, underscoring the critical need for robust and comprehensive coastal management strategies. It is essential to consider and integrate various physical, climatic, and economic factors to make more informed and adaptable decisions in coastal engineering and management. For physical parameters in Dawlish and Aberystwyth, a strong positive correlation indicates that wider dunes are linked to more substantial sea defences, highlighting their vital role in coastal management. Meanwhile, economic parameters across all three study areas suggest that commercial and residential activities are strongly linked to economic value and are key drivers of population growth. Commercial, residential, and population activities are significantly correlated, contributing to overall economic advancement. The methodology adopted in this study to assess coastal vulnerability is multi-dimensional and can be replicated in any suitable area without geographical limitations. Re-evaluating the coastal vulnerability of regions in England and Wales is essential for promoting sustainable development and enhancing the resilience of these coastal areas. This reassessment will be pivotal in shaping future economic and physical growth in these regions. The findings of this research also assist regional and national policymakers in improving or establishing strategies to address the vulnerabilities in coastal areas in England and Wales. Coastal planners can use the results of this study to develop risk management and adaptation measures. These measures can support coastal communities and infrastructure in the event of coastal disasters by implementing either soft or hard engineering solutions.

## Data Availability

Data will be available upon request. Please contact Liuchang Xing (liuchang.xing.21@ucl.ac.uk) to request the data.
